# A framework for estimating cardiorespiratory fitness from diverse exercise tests

**DOI:** 10.1038/s44437-026-00004-3

**Published:** 2026-09-01

**Authors:** Tomas I. Gonzales, Nick Wareham, Soren Brage

**Affiliations:** Institute of Metabolic Science (IMS) Epidemiology, University of Cambridge, Cambridge, UK

**Keywords:** Biomarkers, Cardiology, Health care, Medical research, Physiology

## Abstract

Comparing cardiorespiratory fitness across populations is hindered by unstandardised exercise testing and canonical estimation methods that assume steady-state exercise while applying group-level metabolic equations. We developed a framework that standardises maximal oxygen consumption (V̇O_2_max) estimation by harmonising dynamic heart rate modelling with individual calibration of the metabolic cost of mechanical work, conditioned on characteristics like body size. We evaluated the framework using submaximal and maximal tests (treadmill walking and running, cycle ergometer, stepping, overground walking) from 911 adults. Agreement between framework estimates and directly measured V̇O_2_max was strong (mean bias: 1.5 ml O_2_∙min^−1^·kg^−1^, Pearson’s *r* = 0.80) and remained robust when excluding body size (*r* = 0.76). We then applied the framework and canonical methods to 11,307 UK Fenland Study adults to evaluate associations between estimated V̇O_2_max and cardiometabolic risk. Compared to canonical estimates, framework-estimated V̇O_2_max had stronger associations with a combined cardiometabolic risk profile (blood glucose, fasting insulin, high-density lipoprotein cholesterol) in base (ΔAIC: 3483) and body-size adjusted (ΔAIC: 256) models. The framework standardises fitness estimation across diverse tests, yielding a reliable and comparable biomarker of cardiometabolic health.

## Introduction

Accurate assessment of cardiorespiratory fitness (V̇O_2_max) is crucial for understanding its relationship with cardiometabolic health and mortality in population research^1^. Because direct measurement of V̇O_2_max is often infeasible in large-scale population cohorts, researchers have used a diverse range of submaximal exercise testing modalities and metabolic equations to estimate fitness. As evidenced by various cohort studies, testing protocols vary widely between investigations^2–4^ and among subpopulations within the same cohort^5^. This lack of standardisation creates a barrier to harmonising legacy datasets and restricts the comparability of multicentre trials.

Beyond exercise testing modality differences, the canonical methods used to estimate fitness from these tests can be flawed unless certain physiological assumptions are met. Many methods assume a physiological “steady-state,”^6^ defined here as exercising at a constant intensity for a sufficient duration to allow heart rate and oxygen consumption to reach an equilibrium. Population research, however, frequently uses ramped or continuously increasing exercise protocols where a true steady-state is difficult to achieve, as the exercise heart rate response inherently lags behind the increasing external work rate^7,8^. Canonical methods may also rely on group-level metabolic equations to estimate the metabolic cost of mechanical work^9^. These equations assume that movement economy and biomechanical efficiency do not vary between individuals^10^. Consequently, applying steady-state assumptions and static metabolic equations to dynamic exercise data can introduce substantial measurement error. In epidemiological contexts, this measurement error creates regression dilution bias^5^, which attenuates dose-response relationships required for accurate health risk stratification.

To address this methodological gap, the field requires a test-agnostic approach that does not rely on strict steady-state assumptions or group-level metabolic equations. Here, we present a generalised modelling framework that standardises cardiorespiratory fitness estimation across diverse exercise testing modalities. This framework harmonises foundational principles of exercise physiology^11^ with dynamic modelling of the exercise heart rate response^12–16^. By dynamically modelling the inherently lagged heart rate response and individualising the calibration of the metabolic cost of mechanical work, the framework accounts for non-steady-state exercise and individual physiological variability.

The primary aim of this study was to develop and validate a dynamic, generalised framework for estimating V̇O_2_max across a diverse range of exercise protocols. Secondary aims were to evaluate its criterion validity and physiological plausibility against directly measured values and canonical methods. Finally, to demonstrate its practical epidemiological utility, we applied the framework to the UK-based Fenland study^17,18^ to test the hypothesis that individualising fitness estimation yields unattenuated associations between cardiorespiratory fitness and cardiometabolic risk factors compared to canonical methods.

## Results

**Fig. 1:**
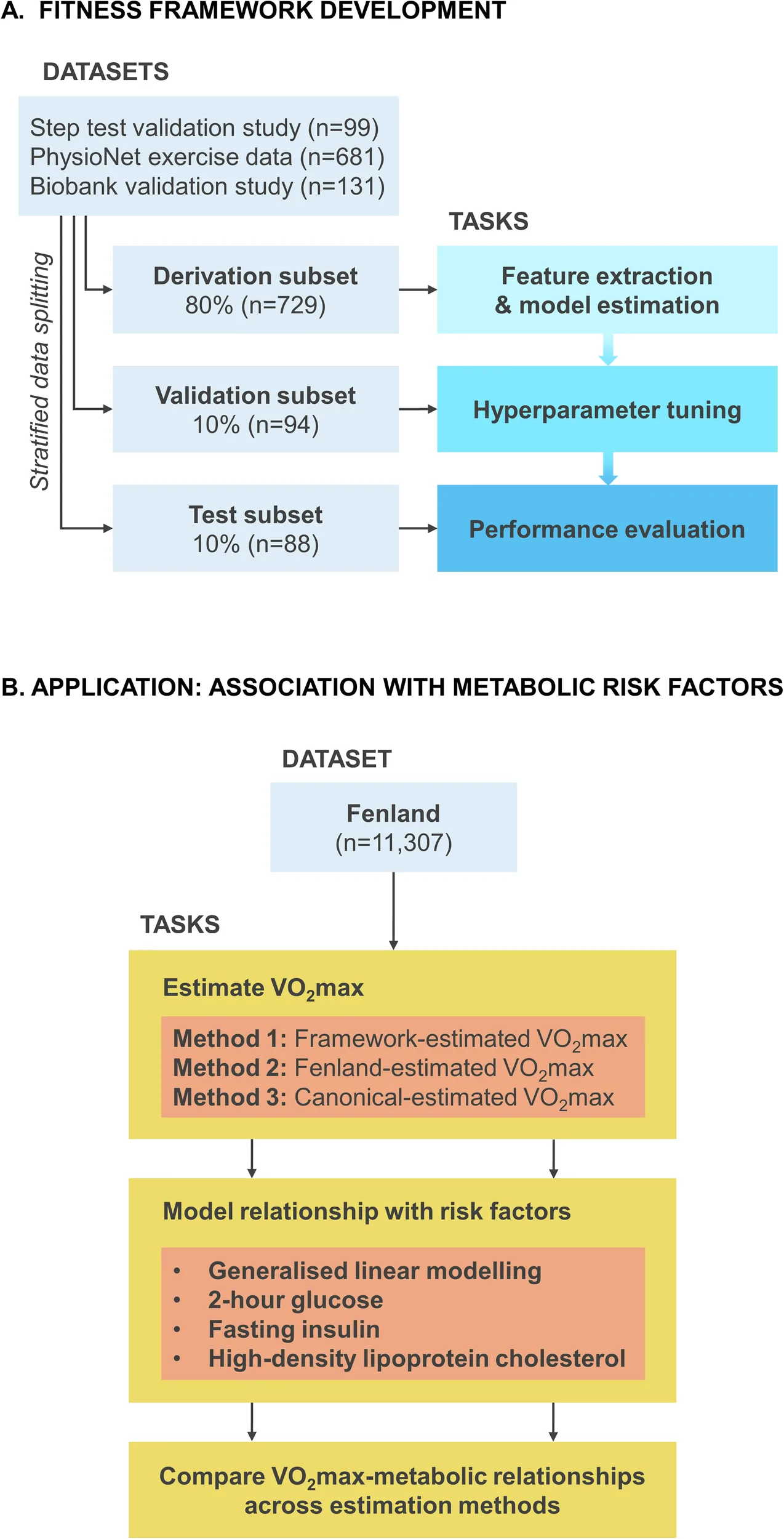
Flow diagram of the study design, comprising two phases. **A** Development and evaluation of a novel framework to estimate V̇O_2_max using exercise test data from several sources; and **B**. Application of the framework to examine associations between estimated V̇O_2_max and metabolic risk in the Fenland Study. V̇O_2_max was estimated using the developed framework and two other approaches based on exercise heart rate response and predicted energy expenditure.

Figure 1 provides a flow diagram of the study design. To develop the fitness estimation framework, we pooled exercise test data from three sources: the Biobank validation study (Cambridge, UK), the Step test validation study (Cambridge, UK), and a repository of exercise test data on PhysioNet (Table 1). The pooled dataset, encompassing a diverse range of exercise modalities and protocols from both submaximal and maximal tests with directly measured V̇O_2_max, was split into derivation (80%), validation (10%), and test (10%) subsets. We used the derivation subset to develop the framework, the validation subset to tune its hyperparameters, and the test subset to evaluate its performance. We then applied the framework to exercise test data from the UK-based Fenland cohort study to examine associations between estimated V̇O_2_max and cardiometabolic risk factors.

**Table 1:** Participant characteristics for datasets included in the framework development phase

Data subset	Derivation (*n* = 729)					Validation (*n* = 94)					Test (*n* = 88)				
Percentile	5th	25th	50th	75th	95th	5th	25th	50th	75th	95th	5th	25th	50th	75th	95th
Age (*y*)	19	25	33	44	60	18	26	32	46	59	20	26	32	47	61
BMI (kg·m^−2^)	20.4	22.6	24.2	25.9	30.0	19.9	22.8	24.1	25.5	29.2	20.5	22.5	24.1	26.5	29.8
Weight (kg)	56	67	74	82	95	57	64	73	79	96	55	66	71	84	97
Height (cm)	160	169	175	180	187	158	167	172	178	186	159	168	172	178	186
Resting HR (bpm)	54	60	61	63	69	50	60	61	63	68	56	61	62	63	72
V̇O_2_max (ml O_2_·min^−1^·kg^−1^)	27.4	37.7	44.2	50.8	58.5	25.9	38.1	44.7	51.7	58.3	26.2	37.2	44.1	51.7	58.9
HRmax (bpm)	161	176	183	190	200	157	174	183	189	201	157	172	181	191	200
Sex (count)															
Females	23.2% (169)					23.4% (22)					22.7% (20)				
Males	76.8% (560)					76.6% (72)					77.3% (68)				

*V̇O*_2_*max* Maximal oxygen consumption, *BMI* Body mass index, *HR* Heart rate. *HRmax* Maximal heart rate. Values are the 5th, 25th, 50th, 75th, and 95th percentiles, unless noted otherwise.

The framework uses three interconnected models to generalise the relationship between the energetic demand of exercise and heart rate dynamics across a wide range of exercise modalities (see “Methods” for a detailed description). Briefly, the framework first standardises exercise intensity from different exercise modalities to oxygen consumption above resting levels (net V̇O_2_). It then uses a linear model to relate net V̇O_2_ to corresponding target heart rate values, representing the heart rate expected for that level of exertion. Finally, in response to instantaneous changes in net V̇O_2_, the framework simulates heart rate dynamics toward these target heart rate values. The framework’s simulation of heart rate dynamics is governed by individual-specific physiological parameters, including V̇O_2_max, HRmax, and metabolic cost coefficients for different exercise modalities. We estimate these parameters for each participant by fitting their measured heart rate data to the framework’s simulated heart rate dynamics using Particle Swarm Optimisation (PSO). The PSO algorithm is guided by an objective function that balances two goals: 1) maximising agreement between simulated and measured heart rate, and 2) ensuring the physiological plausibility of parameter estimates. Physiological plausibility is ensured by minimising the Mahalanobis distance of the estimated parameters from a multivariate normal distribution (MVN) that captures expected physiological relationships based on individual characteristics like age, sex, height, and weight.

**Fig. 2:**
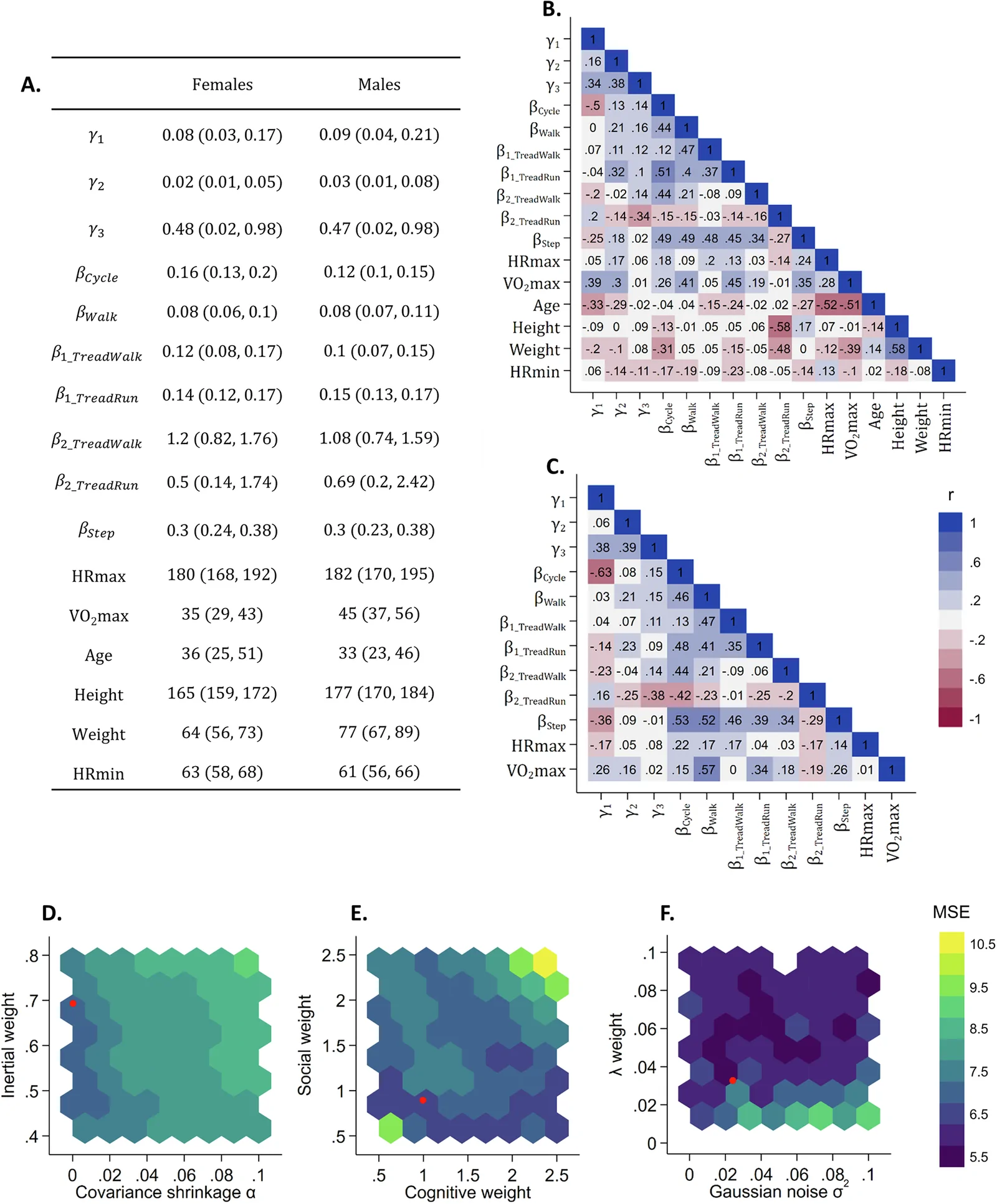
Characterisation and optimisation of the fitness framework. **A** Mean vector and standard deviations (SD) of the multivariate normal distribution (MVN) for males and females. Vector values were estimated in the log space and exponentiated to provide real-world values. Bounds for one SD above and below the mean were also calculated in log space and exponentiated. **B** Unstandardised correlation matrix from the MVN, with cells displaying Pearson’s correlation coefficients. **C** Standardised partial correlation matrix from the MVN, conditional on age, height, weight, and minimum heart rate (HRmin), with cells displaying the partial correlation coefficients. **D**–**F** Heat maps illustrating the relationship between hyperparameter values (inertial weight, social weight, cognitive weight, Mahalanobis distance penalisation weight λ, covariance shrinkage α, Gaussian noise σ) and V̇O_2_max estimation mean squared error (MSE), derived from a heteroskedastic regression meta-model of framework performance. MSE values were predicted for each combination of hyperparameter values across Latin hypercube grid. Red dots represent hyperparameter values that minimise the predicted MSE from the meta-model. Optimised hyperparameter values were then used in the framework application phase of the study. Inertial weight: 0.693, Social weight: 0.895, Cognitive weight: 0.995, λ: 0.328, α: 0.000255, σ: 0.242.

In the derivation dataset, we first fixed V̇O_2_max, HRmax, and HRmin to directly-measured values and then used PSO to estimate the remaining framework parameters. These parameter values, along with individual-level characteristics, were then combined to estimate the sample MVN for computing Mahalanobis distances. Figure 2A displays the estimated mean vector and standard deviations (SD) of the MVN for males and females. Vector values were estimated in the log space and exponentiated to provide real-world values. Bounds for one SD above and below the mean were also calculated in log space and exponentiated. Figure 2B presents the unstandardised correlation matrix from the MVN, with cells displaying Pearson’s correlation coefficients. Figure 2C presents the standardised partial correlation matrix from the MVN, conditional on age, sex, weight, height, and minimum heart rate (HRmin), with cells displaying the partial correlation coefficients. The matrices reveal interrelationships among the framework parameters and differences in the strengths of these relationships upon conditioning.

With the sample MVN estimated, we next used the validation set to optimise parameter estimation for V̇O_2_max. This involved a three-stage process to tune hyperparameters for PSO (inertial, social, and cognitive weights) and MVN regularisation (mean vector Gaussian noise, covariance shrinkage weight). First, we used PSO with Latin hypercube sampling to estimate V̇O_2_max and other framework parameters in the validation set, exploring a wide range of hyperparameter combinations. Mahalanobis distances from the sample MVN guided the search process, ensuring physiological plausibility of parameter estimates. We then constructed a heteroskedastic regression meta-model to predict both V̇O_2_max estimation bias and variance as a function of the hyperparameters and participant characteristics. Finally, we used gradient-based optimisation to minimise the average predicted mean squared error (MSE; squared estimation bias plus variance) from the meta-model, resulting in an optimal hyperparameter configuration. Figure 2D-F presents heatmaps illustrating the relationship between these hyperparameter values and the predicted MSE of V̇O_2_max estimation. Red dots indicate the hyperparameter values that minimise the meta-model’s predicted MSE, which were subsequently used in the framework application phase of the study.

**Fig. 3:**
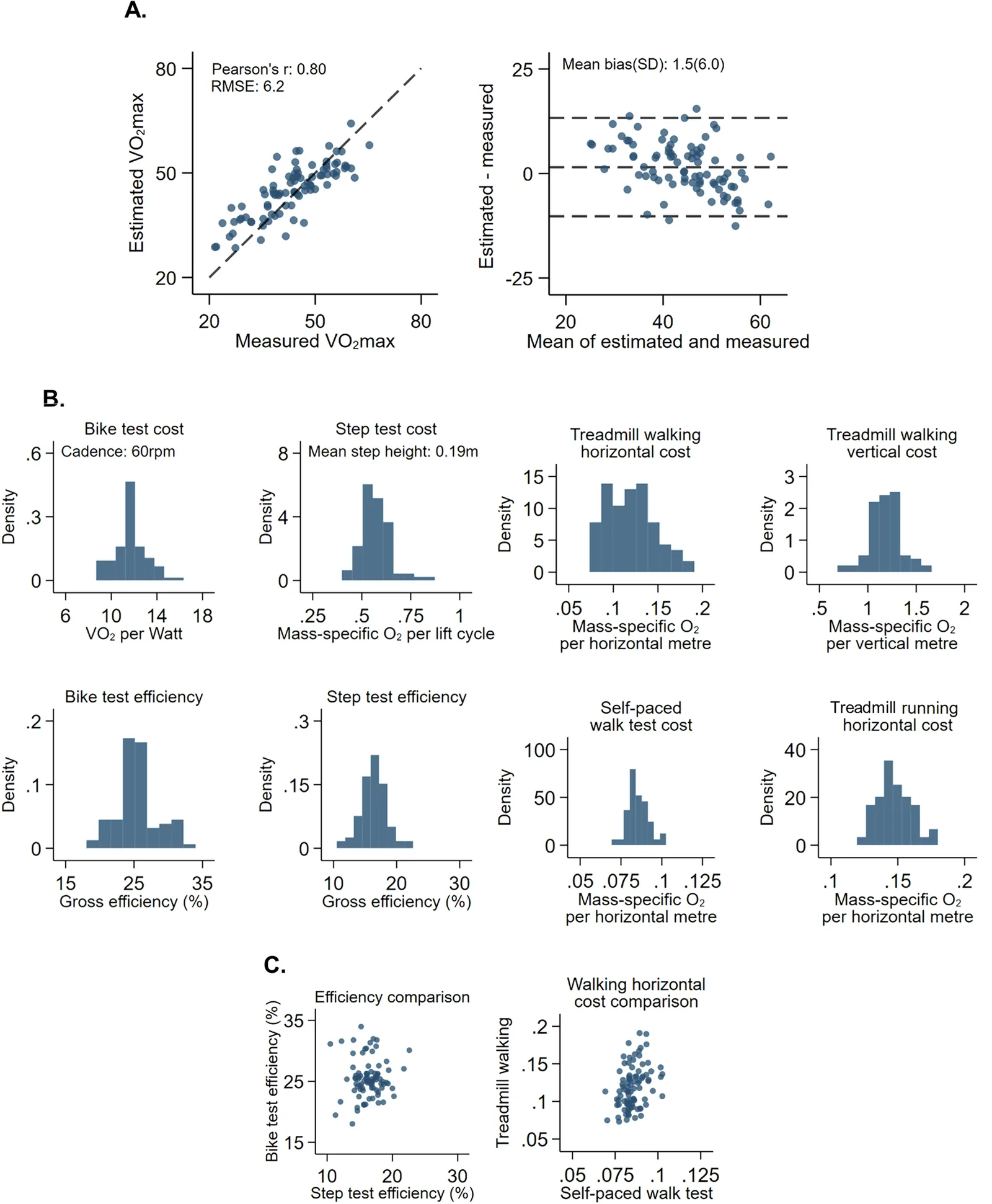
Performance evaluation of the fitness framework. **A** Scatter plots and Bland-Altman plots demonstrating agreement between framework-estimated and directly measured V̇O_2_max. RMSE: Root mean squared error. **B** Histograms of metabolic calibration coefficients estimated by the framework for different exercise modalities. Bike test and step test coefficients have been transformed to facilitate comparison with metabolic coefficients published by the American College of Sports Medicine (ACSM). **C** Scatter plots comparing gross efficiency estimates for bike and step tests, as well as horizontal cost and treadmill and self-paced walking.

Having derived the sample MVN and optimised the hyperparameters, we then evaluated the framework’s performance in the independent test set, held out during derivation and parameter tuning. Figure 3A presents scatter plots and Bland-Altman plots illustrating agreement between framework-estimated and directly measured V̇O_2_max values. Overall mean estimation bias was low, and correlation coefficients were strong (mean bias: 1.5 ml O_2_·min^−1^·kg^−1^; Pearson’s *r*: 0.80, Concordance correlation coefficient: 0.76). Further agreement statistics are reported in Supplementary Table 1, as well as sensitivity analyses to evaluate the framework’s reliance on anthropometric information (height and weight) and close-to-max heart rate data (Supplementary Fig. 1). This involved re-estimating V̇O_2_max without conditioning the MVN on these variables and excluding some heart rate data during the PSO optimisation process. Restricting heart rate data to values below 85% of age-predicted maximum heart rate^19^ slightly reduced agreement (mean bias: 2.0 ml O_2_·min^−1^·kg^−1^; Pearson’s *r*: 0.76). Further reductions in agreement were observed when height and weight were removed (mean bias: 2.3 ml O_2_·min^−1^·kg^−1^; Pearson’s *r*: 0.69).

We conducted a stratified error analysis within the independent test set to investigate estimation error across different physiological profiles. We regressed V̇O_2_max estimation error (framework-estimated minus measured V̇O_2_max) against participant age, sex, BMI, and dataset origin, including cubic terms for age and BMI. Predictive margins from this analysis (Supplementary Fig. 2) demonstrated that the framework exhibited zero V̇O_2_max overestimation bias for middle- and older-aged adults. Minor overestimation bias was observed primarily in younger adults (<30 years) and in individuals with a BMI between ~25 and 33 kg·m^-^^2^. The overall mean overestimation bias in error analysis was 1.5 ml O_2_·min^−1^·kg^−1^ (95% CI: 0.4–2.6).

Figure 3B presents metabolic calibration coefficients estimated by the framework for different exercise modalities. Calibration coefficients were approximately normally distributed and generally similar to values published by ACSM (Table 2)^9^. Estimated gross efficiency for cycle ergometry was 26 ± 3% and for step testing was 15 ± 2%, ranges that are within previously published gross efficiency values (Fig. 3C). The metabolic cost of treadmill walking was 25 ± 8% higher than self-paced walking when performed at similar speeds.

**Table 2:** Comparison of framework-estimated metabolic coefficients with American College of Sports Medicine (ACSM) published values

Metabolic coefficient	Equivalent ACSM published value	Marginal mean (95% CI)
${\beta }_{{Walk}}$	n/a	0.086 (0.084, 0.087)
${\beta }_{{1}_{{TreadWalk}}}$	0.10	0.12 (0.096, 0.14)
${\beta }_{{2}_{{TreadWalk}}}$	1.80	1.18 (1.09, 1.28)**
${\beta }_{{1}_{{TreadRun}}}$	0.20	0.15 (0.14, 0.15)**
${\beta }_{{2}_{{TreadRun}}}$	0.90	0.75 (0.68, 0.82)*
${\beta }_{{Cycle}}$	11.1	11.7 (10.5, 13.0)
${\beta }_{{Step}}$	0.65	0.57 (0.47, 0.67)

β: Exercise modality-specific metabolic coefficients estimated using the framework. Marginal mean values were computed from linear regression models adjusted for sex, age, and body mass index. *ACSM* American College of Sports Medicine. * (*p* < 0.05) and ** (*p* < 0.01) represent statistically significant difference from the corresponding ACSM published value.

**Fig. 4:**
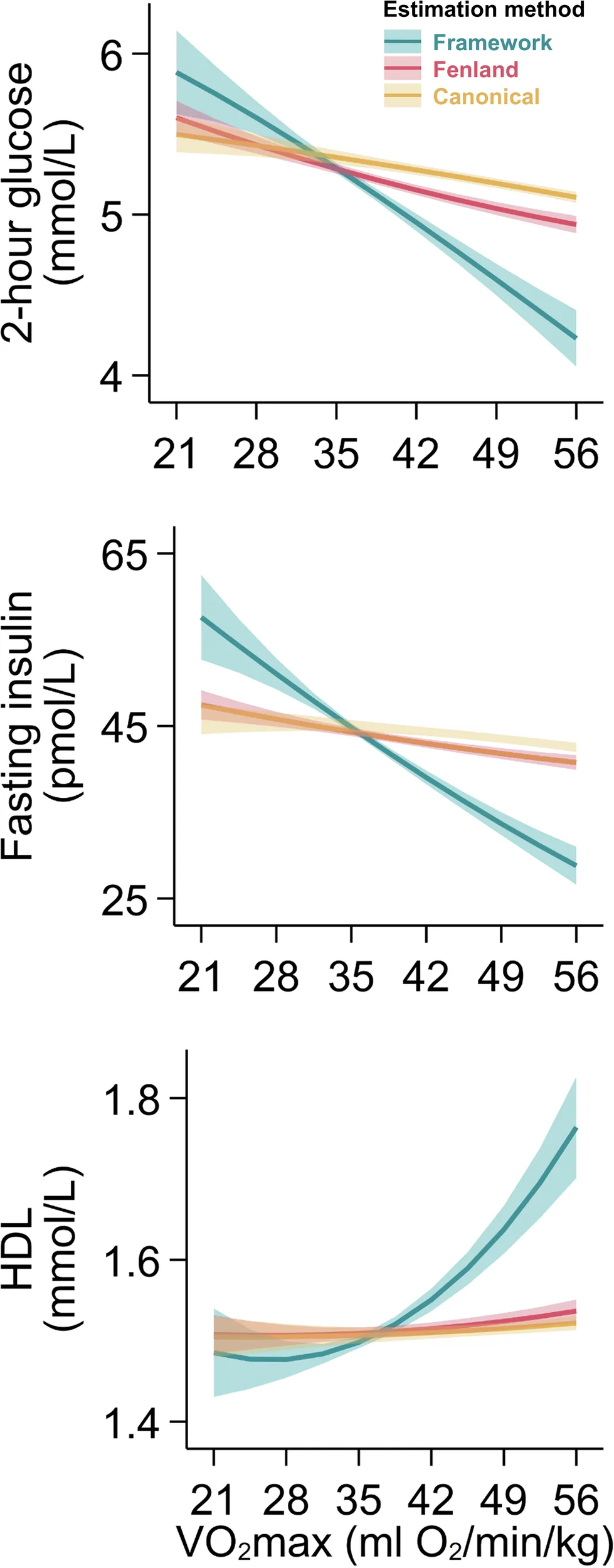
Modelling of cardiometabolic risk. Associations of estimated V̇O_2_max (ml O_2_·min^−1^·kg^−1^) with 2-hour glucose, fasting insulin, and high-density lipoprotein (HDL) cholesterol. Lines represent estimated marginal means and 95% confidence intervals for associations after adjustment for age, sex, resting heart rate, height, and weight.

To demonstrate its construct validity, we applied the framework to the Fenland Study cohort (Table 3) and found that framework-estimated V̇O_2_max had stronger associations with key cardiometabolic risk factors compared to two conventional methods (Fenland-estimated and Canonical-estimated; Table 4). In models adjusted for age, sex, and resting heart rate, one-standard deviation (5.43 O_2_·min^−1^·kg^−1^) higher framework-estimated V̇O_2_max was associated with 10% lower 2 h glucose, 37% lower fasting insulin, and 9.4% higher high-density lipoprotein (HDL) cholesterol. In contrast, one-standard deviation (11.2 ml O_2_·min^-1^·kg^−1^) higher canonical-estimated V̇O_2_max was associated with 4.4% lower 2 h glucose, 12% lower fasting insulin, and 2.6% higher HDL. Fenland-estimated V̇O_2_max associations were between these two sets of estimates. A combined model of all three risk factors showed a substantially better statistical fit for framework-estimated fitness (AIC = 125,743) compared to Fenland-estimated (AIC = 128,849) and Canonical-estimated (AIC = 129,226) fitness. While further adjustment for height and weight attenuated all associations, the framework’s estimates retained the strongest associations with all outcomes (Fig. 4) and maintained a lower AIC compared to conventional estimates (ΔAIC > 200). Median V̇O_2_max estimated by the framework was lower for both females (33.1 ml O_2_·min^−1^·kg^−1^) and males (39.4 ml O_2_·min^−1^·kg^−1^) compared to values from the Fenland-estimated (35.0 and 42.0 ml O_2_·min^−1^·kg^−1^) and Canonical-estimated (43.4 and 51.7 ml O_2_·min^−1^·kg^−1^) approaches.

**Table 3:** Fenland study participant characteristics

Sex	Females (*n* = 5958)	Males (*n* = 5349)
Age (y)	48.2 (42.4–54.0)	48.3 (42.2–54.3)
BMI (kg·m^−2^)	25.2 (22.7–28.7)	26.7 (24.4–29.3)
Weight (kg)	68 (61–78)	84.4 (76.4–93.4)
Height (cm)	164 (160–168)	178 (173–182)
Resting heart rate (bpm)	64 (59–70)	61 (55–67)
2 h glucose (mmol·L^−1^)	5.0 (4.2–5.9)	5.1 (4.2–6.1)
Fasting insulin (pmol·L^−1^)	34.2 (24.2–49–9)	42.8 (29.1–64.1)
HDL cholesterol (mmol·L^−1^)	1.66 (1.41–1.94)	1.30 (1.11–1.53)
Framework–estimated V̇O_2_max (ml O_2_·min^−1^·kg^−1^)	33.1 (30.6–35.9)	39.4 (36.8–42.7)
Fenland–estimated V̇O_2_max (ml O_2_·min^−1^·kg^−1^)	35.0 (30.5–40.3)	42.0 (37.7–49.0)
Canonical–estimated V̇O_2_max (ml O_2_·min^−1^·kg^−1^)	43.4 (37.4–49.2)	51.7 (46.2–57.2)

*BMI* Body mass index, *HDL* High-density lipoprotein cholesterol. Values are median (interquartile range), unless noted otherwise.

**Table 4:** Comparison of V̇O_2_max estimation methods and their association with cardiometabolic risk in the Fenland Study cohort

	Model 1			Model 2		
	Framework-estimated V̇O_2_max	Fenland-estimated V̇O_2_max	Canonical-estimate V̇O_2_max	Framework-estimated V̇O_2_max	Fenland-estimated V̇O_2_max	Canonical-estimated V̇O_2_max
2-h glucose	−0.10 (−0.11, −0.095)	−0.061 (−0.067, −0.054)	−0.044 (−0.050, −0.038)	−0.050 (−0.059, −0.040)	−0.034 (−0.041, −0.027)	−0.026 (−0.031, −0.020)
Fasting insulin	−0.37 (−0.38, −0.36)	−0.17 (−0.18, −0.15)	−0.12 (−0.13, −0.11)	−0.11 (−0.12, −0.087)	−0.041 (−0.053, −0.029)	−0.032 (−0.043, −0.022)
HDL cholesterol	0.094 (0.088, 0.10)	0.036 (0.031, 0.042)	0.026 (0.021, 0.031)	0.022 (0.014, 0.030)	0.0057 (0.0031, 0.011)	0.0058 (0.0010, 0.010)
AIC	125,743	128,849	129,226	123,095	123,307	123,351
ΔAIC		3106	3483		212	256

Coefficients represent the percent difference in each risk factor per 1-standard deviation (SD) difference in V̇O_2_max from generalised linear models (gamma distribution, log link). AIC values are presented as a measure of overall statistical fit across the three risk factors, allowing for a direct comparison between the framework, Fenland-estimated, and Canonical-estimated approaches within each adjustment model.

Model 1: Adjusted for age, sex, and resting heart rate.

Model 2: Adjusted for age, sex, resting heart rate, height, and weight.

*AIC* Akaike’s Information Criterion, *ΔAIC* Difference in AIC relative to framework-estimated V̇O_2_max model, *HDL* High-density lipoprotein cholesterol. Reported values are semi-elastic beta coefficients and 95% confidence intervals for percent difference in each metabolic risk factor per 1-standard deviation (SD) difference in V̇O_2_max. Framework-estimated V̇O_2_max SD: 5.43 ml O_2_·min^−1^·kg^−1^. Fenland-estimated V̇O_2_max SD: 9.60 ml O_2_·min^−1^·kg^−1^. Canonical-estimated V̇O_2_max SD: 11.2 ml O_2_·min^−1^·kg^−1^.

## Discussion

Here we present the development, evaluation, and application of a generalised framework for estimating cardiorespiratory fitness across a wide range of exercise testing modalities and protocols. To our knowledge, this approach is among the first that can be applied to the diversity of exercise tests commonly used in population health research, addressing the persistent challenge of method variability in this field.

The framework allows for individual variation of the metabolic cost of exercise, leading to more precise estimates of V̇O_2_max and other physiological parameters. For example, median metabolic cost coefficients estimated by the framework were closely aligned with group-level metabolic equations published by the ACSM for treadmill walking, cycle ergometry, and step testing^9^. This demonstrates that established principles of exercise physiology are an emergent property of the framework. The notable deviations observed for the ACSM’s coefficients were for the interaction of walking speed and treadmill gradient, as well as treadmill running. These deviations align with previous reports that have also questioned these specific ACSM coefficients^20–22^. This suggests that our framework is not only valid but may even be more robust, as it independently identifies known weaknesses in current field-use equations. Taken together, these findings suggest the framework captures a more fundamental aspect of an individual’s exercise physiology from which established group-level metabolic equations can be derived. Specifically, these individualised coefficients inherently absorb natural variations in substrate oxidation and biomechanical movement economy. While this improves overall precision, it introduces a physiological trade-off in older cohorts, as age-related biomechanical inefficiency may inflate the estimated metabolic cost of exercise. Consequently, for these populations, the resulting V̇O_2_max estimates represent a composite signal of both central cardiorespiratory capacity and peripheral movement economy.

This ability to capture a more fundamental layer of physiology has practical research utility. This is demonstrated by the framework’s associations with cardiometabolic risk factors in the Fenland study. Statistically, framework-estimated V̇O_2_max yielded markedly lower AIC values, indicating that it may explain greater variance in metabolic health than the evaluated methods. Physiologically, these statistics translate into steeper and more pronounced dose-response relationships for 2-h glucose, fasting insulin, and HDL cholesterol. This was observed even when associations were modelled as differences in risk factor per standard deviation differences in estimated V̇O_2_max and adjusting for all covariates used to condition the framework. This performance likely stems from the framework’s ability to capture individual metabolic variation, rather than relying on group-level metabolic equations in conventional methods, thereby mitigating regression dilution bias. Consequently, the framework allows for more accurate stratification of cardiometabolic risk in large cohorts. Furthermore, by providing an individualised, test-agnostic metric, it enables researchers to harmonise fitness data across disparate legacy datasets and multicenter trials.

When applied in the Fenland Study, V̇O_2_max estimates from the framework were lower than those from methods that rely on linear extrapolation from steady-state assumptions. This is not unexpected, as methods that rely on linear extrapolation of the heart rate-to-exercise intensity relationship can overestimate V̇O_2_max from ramped submaximal tests because the continuous increase in workload prevents the achievement of a true physiological steady-state heart rate^5^. Because the exercise heart rate response inherently lags the external work rate, extrapolating this relationship to a presumed maximum heart rate yields an artificially inflated fitness estimate. By dynamically modelling this lagged response without assuming a steady state, our framework’s lower estimates likely mitigate this overestimation bias. Interestingly, this overestimation bias was partially mitigated in canonical models that used a calibrated metabolic cost for ramped exercise, reinforcing the necessity of accurate metabolic modelling^18^. While steady-state and linear extrapolation methods are computationally straightforward to implement, our findings demonstrate that this convenience comes at a cost to physiological accuracy. A primary takeaway for the field is that dynamically modelling the heart rate response and individualising metabolic cost is necessary to avoid attenuating true relationships between fitness and health outcomes.

Other possible applications for the framework include heart rate response to exercise protocols using both progressively increasing and decreasing workloads, as well as daily activities in free-living environments. The framework should mathematically be able to cope with mixed workload protocols, but no such data was available in our dataset. While adapting the framework for consumer wearables in free-living environments is a theoretical future direction, it represents a significant methodological challenge. The intermittent nature of daily physical activity leads to complex heart rate kinetics, and consumer-grade monitors often lack the precision of research-grade equipment^23^. Therefore, the most immediate application of the framework is within field-based epidemiological testing, where it can mitigate the long-standing challenge of method variability.

This study has limitations. Exercise test data used to derive and validate our framework predominantly originate from adults and exclude both children and adolescents. Furthermore, while the framework is designed to be broadly adaptable, the primary formal evaluations in this study utilize cohorts managed by the authorship team. To fully establish its generalisability, future work should focus on validating the framework’s performance in independent, extramural datasets, as well as across diverse global populations and disease groups. Additionally, exercise test data used to construct the framework were predominantly from those with higher fitness levels and contained a higher proportion of males compared to females. It is recognised that this analytic sample is not fully representative of the general population. While our use of multivariate normal regularisation mathematically mitigates the risk of the model overfitting to this specific demographic skew, this sample composition could still impact generalisability when the framework is applied to clinical populations with severe fitness impairments or extreme obesity. Future work should focus on validating the framework’s performance across these specific sub-groups. The data underpinning the framework also does not have cycling at cadences other than 60 rpm and step test data from different step heights from the same individuals. In terms of framework parameter estimation, we used PSO; alternative optimisation methods, such as simulated annealing^24^, genetic algorithms^25^, ant colony optimisation^26^, or a hybrid of these methods, may provide improvements to computational efficiency or scalability. Finally, while the parameters of the dynamical heart rate model have clear mathematical utility, elucidating their precise physiological basis remains an important avenue for future research.

We have presented and validated a generalised framework for estimating fitness from exercise heart rate response. The framework unifies current fitness estimation approaches, offering a versatile and adaptable way to estimate fitness from a wide range of exercise testing modalities and protocols. We demonstrate its application in estimating fitness in a large-scale population study to examine associations with cardiometabolic risk factors. By providing a test-agnostic method for estimating fitness, the framework is positioned to help mitigate the long-standing challenge of method variability in population research. Future studies must validate the framework across diverse demographic groups, extreme fitness phenotypes, and specific clinical populations. Furthermore, to bridge the gap between this methodological development and widespread field adoption, future efforts will focus on developing user-friendly computational packages that will enable the scientific community to easily implement the methods presented in this work.

## Methods

### Exercise test data sources

This study used data from four sources, comprising three for framework development (Biobank validation study, Step test validation study, and a PhysioNet repository) and one for framework application (the Fenland Study). The development datasets included a variety of submaximal exercise tests, such as treadmill, cycle ergometer, self-paced walking, and step tests, and maximal exercise tests with directly measured V̇O_2_max. The application dataset (Fenland) used a submaximal treadmill protocol. Each study had distinct participant recruitment profiles and fitness assessment procedures, which are described in detail in their respective publications and protocol documents. Here we provide a summary of participant inclusion criteria, testing methods, and ethics approval for each study. For the Biobank validation study, ethical approval was obtained from the University of Cambridge Human Biology Research Ethics Committee (Ref: HBREC/2015.16). For the Step test validation study, ethical approval was obtained from the Huntingdon & Peterborough/Fenland Research Ethics Committee (Reg: 07/Q0106/21). For the Fenland study, ethical approval was obtained from the Health Research Authority NRES Committee East of England-Cambridge Central. All participants provided written informed consent. The present study was conducted in accordance with the Declaration of Helsinki. Clinical trial number: not applicable.

### Biobank validation study

#### Background and demographics

Data were originally collected to validate fitness and physical activity assessments in the UK Biobank^5,27^. The current study included data from 131 older adults from the Biobank validation study.

#### Protocol summary

Participants completed the following schedule of exercise tests, conducted over two clinical visits and an inter-visit period: (1) First clinical visit: cycle ergometer testing; (2) Inter-visit period (7–14 days): at-home step test; and (3) Second clinical visit: self-paced walk test and treadmill test. The first clinical visit involved cycle ergometer testing, consisting of a flat test, two ramped tests at different ramp rates, a steady-state test, and another maximal ramped test to elicit V̇O_2_max. Tests were conducted consecutively, separated by at least 15 min of rest, and were specified according to the test that the participant would have been assigned had they been part of UK Biobank^28^.

The target (highest) WR for the second ramped test was at least 30 W greater than the first. Thus, each participant completed a “low” and “high” ramped test. The steady-state test consisted of four incremental 4-minute flat-phases with each work rate increment ranging from 10 to 20 W. All tests ended with a 1-minute recovery-phase where participants sat quietly and motionless on the ergometer. For the ramped max test, participants were fitted with a face mask to measure respiratory ventilation and gas exchange and cycled while work rate increased until exhaustion. V̇O_2_max was considered reached if two of the following criteria were met: a respiratory exchange ratio exceeding 1.20; no V̇O_2_ increase despite increasing work rate (<2.5 ml O_2_·min^−1^·kg^−1^); and no heart rate increase despite increasing work rate. During data analysis, the levelling-off criterion was confirmed by inspecting whether the first differential of heart rate and V̇O_2_ data approached zero over the last 1 min of the maximal test.

During the inter-visit period, participants completed a step test in their home environment while wearing a chest-worn heart rate monitor. Participants were first instructed to find a suitable step between 15 and 23 cm and record the value on an instruction sheet. Then, participants were instructed to rest for 1 min prior to exercise. Participants then began the step test, which consisted of body lifts (one body lift represents a full step-up and step-down cycle) performed over the following stages: (1) 1 min of body lifts at a rate of 15 lifts per minute; (2) 7 min of body lifts with the rate increasing linearly from 15 to 32.5 lifts per minute; and (3) 2 min seated to measure recovery heart rate. The rate of exercise was guided by an audio file defining the stepping cadence. Participants were instructed to rest for 2 min following exercise.

The second clinical visit consisted of a 200 m self-paced walk test and a treadmill test. For the self-paced walk test, two markers were placed 20 m apart in a flat, indoor corridor. Participants then walked at a self-determined pace from one marker to the other and back, completing five laps for a total distance of 200 m. If participants could not complete the total distance, they were instructed to stop walking. After completing the test, participants were provided a chair and asked to sit quietly. Recovery HR was monitored for 1 min. Both the time spent and total distance covered during the test were recorded. The treadmill test was performed according to the following procedure: (1) 3 min of walking at 3.2 km·h^−1^ and 0% incline; (2) 6 min of walking with speed increasing from 3.2 to 5.2 km·h^−1^ at 0% incline; (3) 3 min of walking at 5.2 km·h^-1^ while incline increased from 0 to 6%, followed by 3 min of walking with speed increasing from 5.2 to 5.8 km·h^−1^ and incline increasing from 6 to 10.2%; and (4) 1 min of running with speed increasing from 5.8 to 9.0 km·h^−1^ while incline decreased to 0%, followed by 4.5 min of running with speed increasing from 9.0 to 12.6 km·h^−1^ at 0% incline.

#### Screening criteria

Exclusion criteria were: (1) heart pacemaker or unable to walk without aid; (2) history of angina pectoris; (3) blood pressure greater than 180/110 mmHg; (4) musculoskeletal injury that would impair cycling on the ergometer; (5) pregnancy; and (6) currently taking cardioactive drugs (e.g., beta-blockers, aspirin).

### Step test validation study

#### Background and demographics

Data were originally collected to validate the Cambridge Ramped Treadmill Test from the Fenland study^18,29,30^. The study recruited 51 young adults and 48 older adults.

#### Protocol summary

Participants completed a step test and a maximal treadmill test during a clinical visit. The step test protocol consisted of body lifts (one body lift represents a full step-up and step-down cycle) performed on a 15 or 20 cm step over the following stages: (1) 1 min of body lifts at a rate of 15 lifts per minute; (2) 7 min of body lifts with the rate increasing linearly from 15 to 32.5 lifts per minute; and 3) 2 min seated to measure recovery heart rate. The maximal treadmill test consisted of the following stages: (1) 3 min of walking at 3.2 km·h^−1^ and 0% incline; (2) 6 min of walking with speed increasing from 3.2 to 5.2 km·h^−1^ at 0% incline; (3) 3 min of walking at 5.2 km·h^−1^ while incline increased from 0 to 6%, followed by 3 min of walking with speed increasing from 5.2 to 5.8 km·h^−1^ and incline increasing from 6 to 10.2%; and (4) 1 min of running with speed increasing from 5.8 to 9.0 km·h^−1^ while incline decreased to 0%, followed by running while speed increased from 9.0 km·h^−1^ at 0% incline until exhaustion. V̇O_2_max was considered reached if two of the following criteria were met: a respiratory exchange ratio exceeding 1.20; no V̇O_2_ increase despite increasing work rate (<2.5 ml O_2_·min^−1^·kg^−1^); and no heart rate increase despite increasing work rate.

#### Screening criteria

Inclusion criteria were healthy adults able to walk independently. Exclusion criteria were: (1) contraindications to exercise as measured by the Physical Activity Readiness Questionnaire; (2) any undiagnosed heart condition as determined by resting electrocardiogram (ECG); (3) self-reported breathlessness during limited stair climbing or walking; (4) pregnancy; (5) current reported use of cardioactive medications such as beta-blockers; and (6) systolic and diastolic blood pressure greater than 180/110 mmHg.

### Exercise tests from the University of Malaga Exercise Physiology Lab

#### Background and demographics

We used publicly-available maximal treadmill test data managed by the PhysioNet repository^31^. The database contained 992 maximal treadmill tests conducted from 2005 to 2018 in the Exercise Physiology and Human Performance Lab of the University of Malaga^32,33^. The dataset was used in accordance with the access policy detailed in the PhysioNet Contributor Review Health Data License 1.5.0^34^.

#### Protocol summary

Treadmill test data consisted of a mixture of steady-state and ramped submaximal and maximal protocols, including level walking, graded walking, and running. Heart rate, pulmonary gas exchange, and exercise intensity were continuously measured. Tests were generally structured as follows: (1) An initial stage consisting of 8–10 min of walking at 5.0 km·h^−1^ and 0% incline; and (2) continuous exercise while treadmill speed increased in 0.5 to 1 km·h^−1^ increments. The duration between increments ranged from 2 to 4 min. Participants exercised until there was an increase in V̇O_2_ of less than 2.1 ml O_2_·min^−1^·kg^−1^ between successive increments.

#### Screening criteria

The original dataset was from a retrospective study that included deidentified data from amateur and professional athletes aged 10–63 years. For the present analysis, we excluded pediatric data and used only valid exercise test data performed by adults (*n* = 685).

### Fenland Study

#### Background and demographics

Data were used as the primary application cohort for the framework. The Fenland Study is a prospective cohort of 12,435 participants (born 1950–1975) recruited from Cambridgeshire, UK^17,18^.

#### Protocol summary

Participants performed a submaximal treadmill test consisting of four stages: (1) 3 min of walking at 3.2 km·h^−1^ and 0% incline; (2) 6 min of walking with speed increasing from 3.2 to 5.2 km·h^−1^ at 0% incline; (3) 3 min of walking at 5.2 km·h^−1^ while incline increased from 0 to 6%, followed by 3 min of walking with speed increasing from 5.2 to 5.8 km·h^−1^ and incline increasing from 6 to 10.2%; and (4) 1 min of running with speed increasing from 5.8 to 9.0 km·h^−1^ while incline decreased to 0%, followed by 4.5 min of running with speed increasing from 9.0 to 12.6 km·h^−1^ at 0% incline^35^. Heart rate data were recorded continuously using a chest-worn heart rate monitor^36^. Cardiometabolic health was assessed via blood samples drawn following an overnight fast and 2 h post glycaemic load^37^.

#### Screening criteria

Participant eligibility for exercise testing was assessed using a 12-lead resting ECG, excluding those presenting with unstable angina. In addition, participants who self-reported having a heart condition were examined by a study nurse to determine whether treadmill testing could be conducted safely. Exclusion criteria were: (1) individuals with prevalent diabetes, pregnant or breastfeeding females; (2) those unable to walk unaided for at least 10 min; (3) individuals with psychosis; or (4) those with a terminal illness. 5958 females and 5349 males were included in this analysis after exclusions (Table 3).

### Framework architecture

A flow diagram describing the main components of the framework is shown in Supplementary Fig. 3. The framework takes as input exercise heart rate data and a corresponding, time-varying measure of exercise intensity (e.g., walking speed, treadmill speed and grade, cycling power, stepping frequency and step height). In the present analysis, heart rate and exercise intensity data were interpolated to 1-s resolution, unless they were already recorded at that resolution. No further processing steps were applied to heart rate data, which were already pre-processed using methodologies specific to their original studies. To ensure data integrity despite these varied origins, we implemented a visual screening process prior to inclusion. All heart rate traces were visually inspected to ensure that changes in exercise intensity corresponded with the expected continuous heart rate kinetics and that there was sufficient data coverage across the various stages of a given test. The number of tests included across the studies is provided in Supplementary Table 2.

### Calibration of exercise intensity to net energy expenditure

Group-level metabolic equations can yield imprecise energy expenditure estimates when applied to individual-level exercise test data^35,38^. To address this issue, the first component of the framework uses a system of metabolic equations to individually calibrate exercise intensity from different exercise modalities (e.g., treadmill, cycle ergometer) to net energy expenditure (${\rm{net}}\,{{\rm{VO}}}_{2}$), defined as energy above resting energy expenditure (Supplementary Fig. 4A, B). This approach is similar in principle to previous work that harmonised expressions of energy expenditure across different exercise modalities^35^. The system of equations used in this study was:

1 $$\mathrm{net}{\mathrm{VO}}_{2}=\left\{\begin{array}{l}\mathrm{Walk\; test}:\,{\beta }_{{Walk}}\cdot \mathrm{Speed}\\ \mathrm{Treadmill\; walking}:\mathrm{Speed}\cdot \left({\beta }_{{1}_{{TreadWalk}}}+{\beta }_{{2}_{{TreadWalk}}}\cdot \mathrm{Fractional\; grade}\right)\\ \mathrm{Treadmill\; running}:\mathrm{Speed}\cdot \left({\beta }_{{1}_{{TreadRun}}}+{\beta }_{{2}_{{TreadRun}}}\cdot \mathrm{Fractional\; grade}\right)\\ \mathrm{Cycle\; ergometer\; test}:\,{\beta }_{{Cycle}}\cdot \mathrm{Cycling\; power}\\ \mathrm{Step\; test}:\,{\beta }_{{Step}}\cdot \mathrm{Lift\; power}\end{array}\right.$$

where each equation corresponds to the individualised energetic cost of the indicated exercise modality, and:

- ‘${\rm{net}}\,{{\rm{VO}}}_{2}$’ is oxygen consumption scaled by total-body mass (ml O_2_·min^−1^·kg^−1^)

- ‘Cycling power’ is in watts (W)
- ‘Speed’ is in metres·min^−1^
- ‘Fractional grade’ is treadmill incline percentage expressed in decimal form
- ‘Lift power’ is in J·min^−1^·kg^−1^, computed as the product of step frequency (lifts·min^−1^), step height (metres·lift^−1^), and gravitational acceleration (9.81 m·s^−2^)^39^

The functional forms of the equations nested within ${\rm{net}}\,{{\rm{VO}}}_{2}$ were derived from group-level metabolic equations published by the American College of Sports Medicine (ACSM)^9^. Rather than apply the ACSM group-level metabolic equations, the framework instead individualises this calibration, represented here as beta coefficients (β). This approach allows the framework to account for between- and within-individual differences in the metabolic cost and efficiency of different exercise modalities. We assumed that individual calibration would yield β coefficients that generally align with ACSM group-level metabolic equations. We examine this assumption later in the present analysis (see ‘Assessment of metabolic coefficients’). The derived units of measurement for each β coefficient were:

- ${\beta }_{{Walk}}$: Mass-specific O_2_ cost per horizontal metre (ml O_2_·metre^−1^·kg^-1^)
- ${\beta }_{{1}_{{Tread}}}$: Mass-specific O_2_ cost per horizontal metre (ml O_2_·metre^−1^·kg^−1^)
- ${\beta }_{{2}_{{Tread}}}$: Mass-specific O_2_ cost per vertical metre (ml O_2_·metre^−1^·kg^−1^)
- ${\beta }_{{Cycle}}$: V̇O_2_ per Watt (ml O_2_·min^−1^·kg^−1^·W ^−1^)
- ${\beta }_{{Step}}$: O_2_ cost per unit of mechanical work (ml O_2_·J^−1^)

We specified separate metabolic equations for treadmill walking and treadmill running to account for within-individual differences in the energetic cost of these exercise modalities^40^. The treadmill walking equation was applied when treadmill speeds were less than or equal to 90 metres·min^−1^. The treadmill running equation was applied when treadmill speeds were greater than or equal to 120 metres·min^−1^. These thresholds generally delineate the transition from walking to running in adult human locomotion^41,42^.

The transition from walking to running (e.g., between 90 and 120 metres·min^−1^) was modelled using the following linear interpolation equation for treadmill speed:

2 $${\beta }_{{Transition}}=\frac{{\beta }_{{TreadWalk}}\cdot \left(120-\mathrm{Speed}\right)+{\beta }_{{TreadRun}}\cdot \left(\mathrm{Speed}-90\right)}{120-90}$$

where ${\beta }_{{Transition}}$ is either ${\beta }_{1}$ or ${\beta }_{2}$ in the treadmill test equation and ‘${\rm{Speed}}$’ is treadmill speed. This approach enables the energetic cost of treadmill exercise to be individually calibrated across many potential testing protocols.

### Calibration of net oxygen consumption to target heart rate

Heart rate increases linearly with oxygen consumption and reaches a maximal value when V̇O_2_max is achieved^6^. Based on this principle, the framework uses the following linear equation to calibrate ${\rm{net}}\,{{\rm{VO}}}_{2}$ values to ‘target’ heart rate values (${{\rm{HR}}}_{{\rm{target}}}$):

3 $${\mathrm{HR}}_{\mathrm{target}}=\mathrm{net}\,{\mathrm{VO}}_{2}\cdot \frac{\mathrm{HR}\max }{{\dot{{\rm{V}}}{\rm{O}}}_{2}\max }+\mathrm{HR}\min$$

where:

- HRmin is minimal heart rate
- HRmax is maximal heart rate
- V̇O_2_max is maximal oxygen consumption scaled by total-body mass (ml O_2_·min^−1^·kg^−1^)
- ${{\rm{HR}}}_{{\rm{target}}}$ are heart rate values that correspond to different energy expenditure levels

The ‘HRmax to V̇O_2_max ratio’ can be conceptualised as the oxygen pulse at maximal exercise. The ratio’s value anchors the β coefficients for calibrating exercise intensity to ${\rm{net}}\,{{\rm{VO}}}_{2}$ to values that would allow heart rate and oxygen uptake kinetics to be aligned.

### Modelling of heart rate dynamics

Exercise heart rate response kinetics have historically been modelled using multiple mono-exponential functions for growth and decay^43^. However, within-individual, the time constants for these functions can vary widely when applied to different exercise intensity profiles, making them difficult to interpret across exercise tests^44^. We propose a model of heart rate dynamics where onset and offset heart rate kinetics emerge from the model’s behaviour, rather than functions with fixed time constants (Supplementary Fig. 4C). After calibrating oxygen consumption to corresponding ${{\rm{HR}}}_{{\rm{target}}}$ values, we use a series of coupled differential equations to model heart rate dynamics across the physiological intensity spectrum:

4 $$\Delta {\mathrm{HR}}={{\rm{D}}}_{\mathrm{out}}\cdot \left({\mathrm{HR}}_{\mathrm{target}}-{\mathrm{HR}}_{t}\right)$$

5 $$\Delta {{\mathrm{D}}}_{\mathrm{out}}={C}_{1}\cdot {{\rm{D}}}_{\mathrm{in}}-{C}_{2}\cdot {{\rm{D}}}_{\mathrm{out}}$$

6 $$\Delta {D}_{\mathrm{in}}={C}_{3}\cdot \Delta {{\mathrm{HR}}}_{\mathrm{target}}-{C}_{1}\cdot {{\rm{D}}}_{\mathrm{in}}$$

The first equation models change in heart rate ($\Delta {\mathrm{HR}}$) at time *t* (${{\rm{HR}}}_{t}$) as the difference between ${{\rm{HR}}}_{{\rm{target}}}$ for a given ${\rm{net}}\,{{\rm{VO}}}_{2}$ value and the current heart rate value multiplied by an ‘output drive’ parameter (${{\rm{D}}}_{{\rm{out}}}$). The parameter ${{\rm{D}}}_{{\rm{out}}}$ controls the rate at which instantaneous heart rate approaches a target heart rate value. The second equation uses two factors to model change in the ‘output drive’ parameter (${\Delta {\mathrm{D}}}_{\mathrm{out}}$): a positive ‘input drive’ parameter (${{\rm{D}}}_{{\rm{in}}}$) scaled by ${C}_{1}$ and a negative factor that decays the current value of ${{\rm{D}}}_{{\rm{out}}}$ by ${C}_{2}$. Change in the ‘input drive’ parameter ($\Delta {{\mathrm{D}}}_{\mathrm{in}}$) is also modelled using two factors: a positive factor proportional to $\Delta {{\mathrm{HR}}}_{\mathrm{target}}$ scaled by ${C}_{3}$ and a negative factor that decays the current value of ${{\rm{D}}}_{{\rm{in}}}$ by ${C}_{1}$. The positive and negative factors in the second and third equations (${C}_{1}\cdot {{\rm{D}}}_{\mathrm{in}}$) are equivalent in magnitude.

The values of the scaling parameters (${C}_{1}$, ${C}_{2}$, ${C}_{3}$) within the series of differential equations change dynamically with exercise intensity. These values are determined by linear functions that relate the current heart rate to heart rate reserve:

7 $${C}_{1}={\gamma }_{1}\cdot {\mathrm{HRreserve}}_{\mathrm{fractional}}$$

8 $${C}_{2}=\left(1-{\gamma }_{2}\right)\cdot {\mathrm{HRreserve}}_{\mathrm{fractional}}+{\gamma }_{2}$$

9 $${C}_{3}={\gamma }_{3}\cdot {\mathrm{HRreserve}}_{\mathrm{fractional}}$$

where ${\gamma }_{1}$, ${\gamma }_{2}$, and ${\gamma }_{3}$ are estimated coefficients defining the slope of their respective linear functions and ${{\rm{HRreserve}}}_{{\rm{fractional}}}$ is fractional heart rate reserve:

10 $${{\rm{HRreserve}}}_{{\rm{fractional}}}=\frac{{\rm{HR}}\max -{{\rm{HR}}}_{t}}{{\rm{HR}}\max -{\rm{HR}}\min }$$

${{\rm{HRreserve}}}_{{\rm{fractional}}}$ is a normalised, inverse representation of exercise intensity that ranges from 1 (minimal intensity, HR=HRmin) to 0 (maximal intensity, HR=HRmax). When estimated by the framework, ${\gamma }_{1}$ and ${\gamma }_{2}$ are bound within the interval 0 to 1 and ${\gamma }_{3}$ is strictly positive.

The proposed heart rate dynamics model controls input drive signals caused by changes in energy expenditure during exercise and operating within physiological constraints. This process dynamically regulates the heart’s kinetic profile when changing to a new target heart rate. For example, when approaching the heart’s maximal ventricular beating rate, the model prevents instantaneous heart rate from exceeding this value. Instantaneous heart rate heart is also prevented from falling below a minimal value.

### Estimation of model parameters

The framework consists of three interconnected models, each with its own set of parameters:

- Calibration of exercise intensity to net energy expenditure: ${\beta }_{{Walk}}$, ${\beta }_{{1}_{{Tread}}}$, ${\beta }_{{2}_{{Tread}}}$, ${\beta }_{{Cycle}}$, ${\beta }_{{Step}}$
- Calibration of net energy expenditure to target heart rate: V̇O_2_max, HRmax, HRmin
- Heart rate dynamics: ${\gamma }_{1}$, ${\gamma }_{2}$, ${\gamma }_{3}$

The goal of parameter estimation is to identify the set of parameter values that best fits the framework’s simulation of heart rate dynamics to measured exercise heart rate data. This process involves these heart rate measurements, exercise intensity data, and individual-level characteristics to estimate the parameters listed above.

### Parameter estimation using particle swarm optimisation

Due to the novel architecture of the framework, we used Particle Swarm Optimisation (PSO), a metaheuristic algorithm for complex optimisation problems (Supplementary Fig. 5)^45^. PSO guides the behaviour of a ‘swarm’ of particles, where each particle represents a vector of parameter values intended to optimise the model. Particles explore the parameter space and iteratively adjust their trajectories based on a specified objective function (described in detail below). By sharing information about their positions and performance with other particles, the swarm gradually converge towards an optimal set of parameters. The particle that achieves the best objective function value upon termination of the algorithm is selected as the final estimated parameter set.

In our implementation, each PSO instance used 64 particles. Initial parameter values for each particle were randomly sampled from uniform distributions within predefined ranges (Supplementary Table 3). The algorithm was terminated after 64 iterations, a value chosen to balance computational efficiency with sufficient exploration of the parameter space. Particle movement and search behaviour were controlled by three hyperparameters: inertial weight, social weight, and cognitive weight. These hyperparameters control the influence of a particle’s own best position (determined by its objective function value) and the swarm’s best position on the particle’s trajectory. The values for these hyperparameters were determined as described in section ‘Tuning of framework hyperparameters’. A ring topology was used for the communication architecture, with each particle sharing its position with its two nearest neighbours. This topology was chosen because it promotes exploration of the parameter space and helps prevent premature convergence to a suboptimal solution^46^.

### Objective function with Mahalanobis distance penalty for physiological plausibility

We specified the PSO objective function, denoted $f\left(x\right)$, to find a particle parameter vector x that satisfies two criteria: (1) agreement between modelled and observed heart rate data; and (2) adherence to physiological constraints. This is achieved through a two-component objective function. The first component maximises a composite coefficient of determination:

11 $$\frac{1}{2}\left({{\rm{R}}}_{{\rm{overall}}}^{2}+{{\rm{R}}}_{{\rm{within}}}^{2}\right)$$

where:

- ${{\rm{R}}}_{{\rm{overall}}}^{2}$ is the overall coefficient of determination, which quantifies the proportion of variance in the measured heart rate response explained by the modelled response
- ${{\rm{R}}}_{{\rm{within}}}^{2}$ is the within-exercise-intensity coefficient of determination, which quantifies agreement between measured and modelled heart rate response at each distinct ‘target’ heart rate level

If only ${{\rm{R}}}_{{\rm{overall}}}^{2}$ is used, the shapes of the measured and modelled heart rate response do not agree. Using only ${{\rm{R}}}_{{\rm{within}}}^{2}$ results in an offset between modelled and measured heart rate responses, despite their shapes agreeing. Defining the first component as the average of ${{\rm{R}}}_{{\rm{overall}}}^{2}$ and ${{\rm{R}}}_{{\rm{within}}}^{2}$ results in good overall agreement and shape correspondence between the measured and modelled heart rate responses.

While the first component of the objective function ensures a good fit to observed heart rate data, it does not guarantee that the resulting model parameters are always physiologically plausible. To address this, the objective function incorporates a penalty term that encourages solutions consistent with known physiological relationships between model parameters and individual characteristics. For example, younger, normal-weight adults tend to exhibit higher V̇O_2_max values and faster heart rate kinetics compared to older, obese adults. The penalty term is based on the Mahalanobis distance, denoted ${d}_{M}$, which measures how far a given solution deviates from expected physiological norms (Supplementary Fig. 6). Assuming these parameters and characteristics are jointly normally distributed in the population, ${d}_{M}$ is computed using an underlying population multivariate normal (MVN) distribution, denoted ${\mathscr{N}}\left(\mu ,\Sigma \right)$:

12 $${d}_{M}=\sqrt{\left(x-\mu \right)\cdot {\Sigma }^{-1}\cdot {\left(x-\mu \right)}^{{\prime} }}$$

where:

- ${d}_{M}$ is the Mahalanobis distance between a PSO particle parameter vector (x) from expected physiological norms, conditioned on individual-level characteristics
- μ is the population mean vector of the MVN, containing model parameters and individual-level characteristics
- Σ is the population covariance matrix of the MVN, describing interrelationships between framework parameters and individual-level characteristics.

The penalty term in the objective function is then defined as:

13 $$\lambda \cdot {d}_{M}$$

where λ is the physiological plausibility weight hyperparameter that controls the influence of ${d}_{M}$ on the optimisation process. Combining the first component of the objective function and the penalty term yields the following overall objective function $f\left(x\right)$:

14 $$f\left(x\right)=\frac{1}{2}\left({{\rm{R}}}_{\mathrm{overall}}^{2}+{{\rm{R}}}_{\mathrm{within}}^{2}\right)-\lambda \cdot {d}_{M}$$

Defining the objective function as $f\left(x\right)$ encourages the PSO to find solutions that maximise agreement between measured and modelled heart rate data while minimising the Mahalanobis distance from expected physiological norms. Appropriate tuning of λ is required to balance the trade-off between model fit and physiological plausibility.

Using the Mahalanobis distance, ${d}_{M}$, as a penalty term allows for the estimation of individual-calibration parameters (β), even when data for a specific exercise modality are missing (i.e., the individual did not perform that exercise). While each PSO particle represents a complete set of parameters for all modalities, ${d}_{M}$ is calculated using only the parameters corresponding to modalities performed by a given individual. However, because the MVN distribution encodes relationships between individual-level characteristics and all parameters, minimising ${d}_{M}$ effectively constrains the values of unobserved parameters by conditioning them on observed parameters through the MVN’s covariance structure. In this way, prior information encoded in the MVN distribution is used to inform the estimation of parameters for which data are unavailable.

### Regularisation of Mahalanobis distance penalty

In practice, the underlying population MVN ${\mathscr{N}}\left(\mu ,\Sigma \right)$ is unknown. Its parameters μ and Σ are instead estimated from a sample, yielding a sample-estimated MVN ${\mathscr{N}}\left(\hat{\mu },\hat{\Sigma }\right)$ with sample mean vector $\hat{\mu }$ and sample covariance matrix $\hat{\Sigma }$. These are used to compute the Mahalanobis distance ${d}_{M}$ in lieu of the population MVN:

15 $${d}_{M}=\sqrt{\left(x-\hat{\mu }\right)\cdot {\hat{\Sigma }}^{-1}\cdot {\left(x-\hat{\mu }\right)}^{{\prime} }}$$

However, a sample-estimated MVN may be misspecified and overfit to the data it was derived from, leading to poor generalisation and overconfident estimates of ${d}_{M}$. To mitigate the risk of the PSO overfitting to potentially spurious characteristics of a sample, we regularise the sample-estimated MVN by applying Gaussian noise to elements in $\hat{\mu }$ that correspond to individual-level characteristics:

16 $$\hat{\mu }\leftarrow \hat{\mu }+{\mathscr{N}}\left(0,{\sigma }^{2}\right)$$

where:

- ${\mathscr{N}}\left(0,{\sigma }^{2}\right)$ is zero-mean Gaussian noise with variance hyperparameter ${\sigma }^{2}$
  Furthermore, we also regularise the sample-estimated MVN by applying covariance shrinkage to $\hat{\Sigma }$:
  17 $$\hat{\Sigma }\leftarrow \left(1-\alpha \right)\cdot \hat{\Sigma }+\alpha \cdot \frac{{tr}\left(\hat{\Sigma }\right)}{p}\cdot {I}_{p}$$
  where:
- α is the shrinkage intensity hyperparameter
- ${tr}\left(\hat{\Sigma }\right)$ is the trace of $\hat{\Sigma }$
- p is the number dimensions in $\hat{\Sigma }$
- ${I}_{p}$ is the p x p identity matrix

Adding Gaussian noise to the individual-level characteristics in $\hat{\mu }$ introduces uncertainty. This reduces the risk of overfitting to potentially sample-specific characteristics. Covariance shrinkage pulls $\hat{\Sigma }$ towards a scaled identity matrix, which has two primary benefits^47^. First, it dampens the strength of estimated associations between parameters, reducing the influence of spurious correlations on ${d}_{M}$ calculations. Second, it preserves the positive-definiteness of $\hat{\Sigma }$, a necessary property for calculating ${d}_{M}$. Appropriate tuning of ${\sigma }^{2}$ and α are required to balance the benefits of regularisation while retaining sufficient information within the sample-estimated MVN.

### Model training

Each dataset (e.g. Biobank validation study, Step test validation study, PhysioNet treadmill exercise dataset) was split into derivation (80%), validation (10%), and test (10%) subsets using stratified sampling by age, weight, and sex. Derivation, validation, and test subsets were then pooled, respectively, for model training and evaluation.

### Estimation of sample covariance matrix for Mahalanobis distance calculations

In the derivation set, we first fixed V̇O_2_max, HRmax, and HRmin to their directly measured values. This ensured that relationships between these parameters, other estimated model parameters, and individual-level characteristics would be accurately reflected in the estimated sample MVN. We then used PSO to estimate values for all other framework parameters. For this process, PSO hyperparameters for inertial and social weight were each set to 1.3 and cognitive weight was set to 0.6. Only the first component of the objective function was applied, as the sample MVN had not been estimated thus far and could not be used to compute ${d}_{M}$. Following this process, we defined a parameter vector for each participant that included the estimated parameters, directly measured values for V̇O_2_max, HRmax, and HRmin, and individual-level characteristics related to fitness (age, height, weight, sex). The parameter vectors were then combined into a single dataset.

To estimate the sample MVN, we applied the maximum-likelihood expectation-maximisation algorithm to the combined dataset^48^. For this approach, V̇O_2_max, HRmax, HRmin, estimated model parameters, and continuous individual-level characteristics (age, height, weight, resting heart rate) were entered as the continuous dependent variables constituting the dimensions of the MVN. Because binary variables violate the assumption of multivariate normality, sex was entered as an independent categorical covariate. Consequently, the resulting mean vector $\hat{\mu }$ was conditioned on sex, while the resulting pooled covariance matrix $\hat{\Sigma }$ represented the conditional covariance of the continuous variables.

We chose expectation-maximisation because it allowed us to utilise all participant data, including those missing parameters due to the pooling of datasets with different exercise testing protocols. We acknowledge that applying expectation-maximisation in this context violates the missing at random assumption, as the missingness is related to study origin. This could potentially introduce bias into the sample MVN. However, we opted for expectation-maximisation over a complete-case analysis to maximise the use of available data, especially given the regularization techniques described earlier that would be applied to mitigate potential bias in the sample MVN.

### Tuning of framework hyperparameters

We used a three-stage hyperparameter optimization strategy to maximise agreement between estimated and directly-measured V̇O_2_max. First, using the sample MVN from the derivation set, we applied PSO to estimate framework parameters in the validation set across a dense grid of hyperparameter value combinations. Grids were generated using Latin hypercube sampling, a space-filling design that efficiently explores the parameter space^49^. For each participant, 512 unique hyperparameter combinations were generated, and three replicate estimations were performed for each combination to account for the stochastic nature of the PSO algorithm. We then evaluated framework performance for each combination as the difference between estimated and directly-measured V̇O_2_max (e.g. estimation bias). This yielded a dataset of relationships between hyperparameters, participant characteristics, and framework performance.

Next, we constructed a heteroskedastic regression meta-model to predict both estimation bias and its variance as a function of the hyperparameters and participant characteristics. The meta-model served as a computationally efficient surrogate for the complex relationships between hyperparameters and model performance, allowing us to explore a wider range of hyperparameter values without repeatedly running the full PSO estimation. Importantly, heteroskedastic regression enables modelling of non-constant variance in estimation bias across different hyperparameter configurations, allowing the Mean Squared Error (MSE) of the framework to be computed directly. This allows for the identification of hyperparameters that optimally balance bias and variance, minimising the overall error. To capture potential non-linearities and heterogeneity in the bias-hyperparameter relationships, the meta-model incorporated main effects and all two-way interactions between hyperparameters as well as interactions with participant age and BMI.

Finally, to identify the optimal hyperparameter values, we defined a meta-objective function that calculated the average predicted MSE from the meta-model across a grid of age and BMI values. Newton–Raphson’s method was then used to minimise the meta-objective function, resulting in a hyperparameter configuration that minimised the average MSE of the framework across a range of individual characteristics.

### Model evaluation

#### Assessment of agreement

Using the sample MVN from the derivation set and optimal hyperparameter values identified in the validation set, we applied the framework to the test set to estimate both V̇O_2_max and metabolic cost coefficients. These values were estimated by fitting each participant’s measured heart rate to the framework’s simulated heart rate dynamics, as described previously. To evaluate agreement between framework-estimated and directly-measured V̇O_2_max, we used scatter plots and Bland–Altman plots and computed the following metrics:

- Mean bias error ($\bar{\varepsilon }$), calculated as the average difference between measured and estimated values.
- Standard deviation of the mean error (${\sigma }_{\bar{\varepsilon }}$).
- Root mean squared error (RMSE, calculated as $\sqrt{{\bar{\varepsilon }}^{2}+{{\sigma }_{\bar{\varepsilon }}}^{2}}$)
- Pearson’s correlation coefficient (r)
- Concordance correlation coefficient (${\rho }_{c}$), quantifies agreement by considering both correlation and bias^50^.
- Bias correction factor (${C}_{b}$, calculated as ${\rho }_{c}/r$), quantifies the deviation of the best-fit line from the line of perfect concordance (45° line). ${C}_{b}$ values closer to 1 indicate minimal bias.

As a sensitivity analysis, we reapplied the framework to the test set under different data restriction scenarios: when not using weight and height to condition the MVN during parameter estimation, and when censoring data greater than 85% age-predicted maximal heart rate. These scenarios were explored to examine the framework’s reliance on both anthropometric data and heart rate data closer to maximal values. To investigate the distribution of estimation error across different physiological profiles, we conducted a stratified error analysis within the test set. We regressed V̇O_2_max estimation error (framework-estimated minus measured V̇O_2_max) against participant age, sex, BMI, and dataset origin, including cubic terms for age and BMI. Predictive margins and 95% confidence intervals were computed to evaluate how V̇O_2_max estimation error varied across continuous ranges of age and BMI.

### Assessment of metabolic coefficients

We examined the assumption that the framework would yield individual-calibration parameters (β coefficients) that correspond to group-calibration coefficients published by ACSM. We used regression analysis to summarise β coefficient estimates for each exercise modality and compared their marginal mean values with ACSM group-level metabolic equations^9^. For reference, the coefficients in the ACSM equations are:

- Treadmill walking (horizontal component): 0.1 ml O_2_·metre^−1^·kg^−1^
- Treadmill walking (vertical component): 1.8 ml O_2_·metre^−1^·kg^–1^
- Treadmill running (horizontal component): 0.2 ml O_2_·metre^−1^·kg^−1^
- Treadmill running (vertical component): 0.9 ml O_2_·metre^−1^·kg^−1^
- Stepping (0.19 metre step height per lift cycle): 0.65 ml O_2_·lift^−1^·kg^−1^
- Cycle ergometry (60 rpm cadence): 11.1 ml O_2_·min^−1^·W^−1^

To facilitate comparison with the ACSM cycle ergometry coefficient, ${\beta }_{{Cycle}}$ (ml O_2_·min^−1^·kg^−1^·W^−1^) was multiplied by total-body mass. Similarly, ${\beta }_{{Step}}$ (ml O_2_·J^−1^) was multiplied by gravitational acceleration (9.81 m·s^−^^2^) and step height per lift cycle (metres·lift^−1^) to facilitate comparison with the ACSM stepping coefficient. The mean (range) step height used for step testing in the present analysis was 19 (15–20) cm. ACSM does not publish a metabolic equation for self-paced walking, but the metabolic cost of treadmill walking is reported to be greater than self-paced walking^51^. We compared estimated ${\beta }_{{Walk}}$ with ${\beta }_{{TreedWalk}}$ to examine the percent difference in metabolic cost between modalities.

Since ${\beta }_{{Cycle}}$ and ${\beta }_{{Step}}$ each incorporate direct measurements of mechanical work, gross efficiency can be estimated for cycle ergometry and step testing. Gross efficiency is the ratio of mechanical work produced during exercise to the total metabolic energy expenditure required to produce that work. Assuming a caloric equivalent of oxygen of 20.35 J·ml O_2_^−1^^52^, gross efficiency for cycle ergometry (${E}_{{Cycle}}$) was computed as:

18 $${E}_{{Cycle}}=\frac{100 \% }{{\beta }_{{Cycle}}\cdot 20.35\left({\rm{J}}/\mathrm{ml}{{\rm{O}}}_{2}\right)\cdot \mathrm{Weight}(\mathrm{kg})}\cdot 60{\rm{s}}/\min$$

where ‘Weight’ is total-body mass. Gross efficiency for step testing (${E}_{{Step}}$) was computed as:

19 $${E}_{{Step}}=\frac{100 \% }{{\beta }_{{Cycle}}\cdot 20.35\left({\rm{J}}/\mathrm{ml}{{\rm{O}}}_{2}\right)}$$

Gross efficiency values for cycle ergometry and step testing were compared within-individual and with previously reported ranges^53,54^. When performing cycle ergometry at a fixed 60 rpm cadence, ${E}_{{Cycle}}$ can range from 20% to 30%. ${E}_{{Step}}$ can range from 10% to 20% at a step height of approximately 0.2 metres.

### Comparative analysis of associations with cardiometabolic risk

To assess the clinical utility of our framework and compare its performance against established methods, we estimated V̇O_2_max from exercise test data in the Fenland Study using three methods: (1) our novel framework; (2) a study-specific linear regression method previously developed for the Fenland study (Fenland-estimated)^29^; and (3) a canonical linear regression method based on ACSM equations (Canonical-estimated)^9^. Both the Fenland-estimated and Canonical-estimated approaches extrapolate the linear relationship between exercise heart rate response and exercise intensity to age-predicted maximal HR to estimate V̇O_2_max^6^. We then examined associations between each V̇O_2_max estimation method and three cardiometabolic risk factors: 2-hour glucose, fasting insulin, and high-density lipoprotein (HDL) cholesterol. These were chosen because they are indicators of distinct cardiometabolic health pathways (e.g., glycaemic control, insulin resistance, lipid metabolism) with known relationships with fitness^55,56^.

Because cardiometabolic risk factors are physiologically related, we used a structural equation modelling approach to simultaneously estimate and compare the association between each fitness estimate and the three risk factors. This approach allows for the evaluation of each fitness measure against the complete metabolic risk profile, rather than treating each biomarker in isolation. This yields a single measure of model fit (Akaike Information Criterion; AIC) for direct comparison. Within the structural equation model, the relationship for each risk factor was specified by a generalised linear model with a Gamma distribution and a log link function. To account for potential non-linear relationships, quadratic terms for V̇O_2_max were included. The analysis for each fitness estimation method was conducted under two levels of progressive covariate adjustment: (1) adjusted for age, sex, and resting heart rate; and (2) additionally adjusted for height and weight. The overall fit for each method and adjustment level was then compared using the AIC values, where lower values indicate a superior fit. Associations were also visualised by plotting the estimated marginal means of each risk factor across a range of V̇O_2_max values.

### Statistical software

Statistical analyses were performed with Stata (Version 18; StataCorp, College Station, TX). A *p*-value of 0.05 or less was considered statistically significant. All code to implement the modelling framework was written in Mata, a C-like matrix programming language that is a component of Stata.

## Supplementary information

Supplements

## Data Availability

Biobank validation study, Step test validation study, and Fenland study data are available from University of Cambridge IMS Epidemiology upon reasonable request: https://studies.epi.ims.cam.ac.uk/data-sharing. These datasets were acquired by making an internal data request to the University of Cambridge IMS Epidemiology data management team. Treadmill data from PhysioNet are available at the following open-access repository: https://physionet.org/content/treadmill-exercise-cardioresp/1.0.1/. This dataset was acquired via direct download from the repository.

## Code availability

Study code is available upon reasonable request to the corresponding author.
