# Supplementary material for: A framework for estimating cardiorespiratory fitness from diverse exercise tests
Source: Npj Exerc Med Health. 2026 Sep 1;1(1):4. doi: 10.1038/s44437-026-00004-3 (PMC13561033; doi:10.1038/s44437-026-00004-3)

A framework for estimating cardiorespiratory fitness from diverse exercise tests

Tomas I. Gonzales, Nick Wareham, & Soren Brage

Affiliations

Institute of Metabolic Science (IMS) Epidemiology, University of Cambridge, Cambridge, UK

SUPPLEMENTARY MATERIALS

Corresponding author

Tomas I. Gonzales, PhD

IMS Epidemiology

Level 3 Institute of Metabolic Science

University of Cambridge School of Clinical Medicine

Cambridge, UK, CB2 0SL

Email: tg421@cam.ac.uk

ORCID: 0000-0003-0085-8771

Supplementary Table 1

Agreement statistics for framework-estimated and directly measured $\dot{V}O_2max$ (ml $O_2 \cdot min^{-1} \cdot kg^{-1}$) under different data-restriction scenarios. When applying weight and height restrictions, these variables were not used when conditioning the multivariate normal distribution during parameter optimisation. When applying heart rate data restrictions, heart rate greater than 85% age-predicted max heart rate were censored prior to fitting the framework. Age-predicted max heart rate was computed as: 208-0.7·Age(years)

Restrictions applied when fitting the framework	$\bar{\varepsilon}$	$\sigma_{\bar{\varepsilon}}$	RMSE	r	ρ_c	C_b
No data restrictions applied	1.5	6	6.2	0.80	0.76	0.95
Weight and height	2.1	6.6	6.9	0.76	0.69	0.91
HR greater than 85% age-predicted max HR	2	6.7	7	0.76	0.73	0.97
HR greater than 85% age-predicted max HR, weight, and height	2.3	7.5	7.8	0.69	0.66	0.96

HR: measured heart rate $\bar{\varepsilon}$: Mean error, $\sigma_{\bar{\varepsilon}}$: Standard deviation of the mean error, RMSE: Root mean squared error, r: Pearson's correlation coefficient, ρ_c: Concordance correlation coefficient, C_b: Bias correction factor

Supplementary Table 2

Tabulation of exercise test modalities across all included studies.

	Biobank validation	Step test validation	Exercise tests from the University of Malaga Exercise Physiology Lab
Cycle ergometry test			
High ramp rate	131		
Low ramp rate	130		
Steady-state	131		
Maximal	131		
Step test	106	99	
Treadmill test	130	99	681
Self-paced walk test	129		

Supplementary Table 3

Uniform distribution bounds for Particle Swarm Optimisation (PSO) particle initialisation, which defined the range from which each particle's initial position in the parameter space was sampled.

Parameter	Lower bound	Upper bound
γ_1	0.1	0.5
γ_2	0.01	0.10
γ_3	0.05	0.25
β_{Walk}	0.05	0.15
$\beta_{1_{TreadWalk}}$	0.05	0.15
$\beta_{2_{TreadWalk}}$	1	2
$\beta_{1_{TreadRun}}$	0.15	0.25
$\beta_{2_{TreadRun}}$	0.5	1.5
β_{Cycle}	0.1	0.2
β_{Step}	0.1	0.5
$\dot{V}O_2max$	15	75
HRmax	120	200

Supplementary Figure 1

Sensitivity analysis of the framework's reliance on demographic information. Scatter plots and Bland-Altman plots demonstrate agreement between framework-estimated and directly measured $\dot{V}O_2max$ (ml $O_2 \cdot min^{-1} \cdot kg^{-1}$) under different data-restriction scenarios. When applying weight and height restrictions, these variables were not used when conditioning the multivariate normal distribution during parameter optimisation. When applying heart rate data restrictions, heart rate greater than 85% age-predicted max heart rate were censored prior to fitting the framework. Age-predicted max heart rate was computed as: 208-0.7·Age(years).

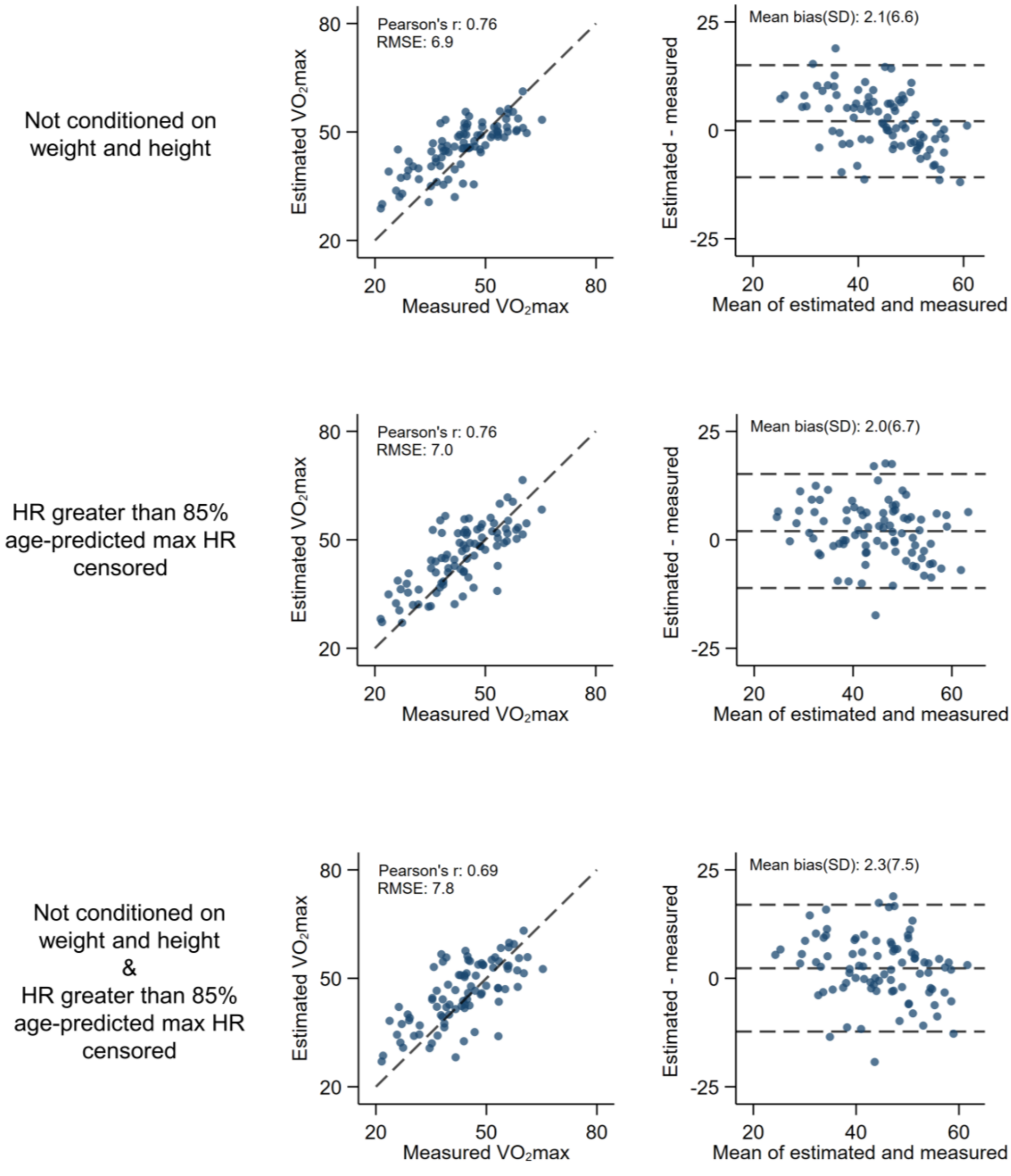

Supplementary Figure 2

Predictive margins of $\dot{V}O_2$max estimation bias by age and body mass index (BMI). Solid lines represent the predicted mean estimation error (framework-estimated minus directly measured $\dot{V}O_2$max) across continuous ranges of age (Top) and BMI (Bottom) within the independent test set. Predictive margins were derived from a regression model adjusting for participant age, sex, BMI, and dataset origin (Biobank validation, Step test validation, Malaga). Polynomial terms for age and BMI were included to capture non-linear relationships. The horizontal dashed line represents zero estimation bias. Shaded regions represent 95% confidence intervals.

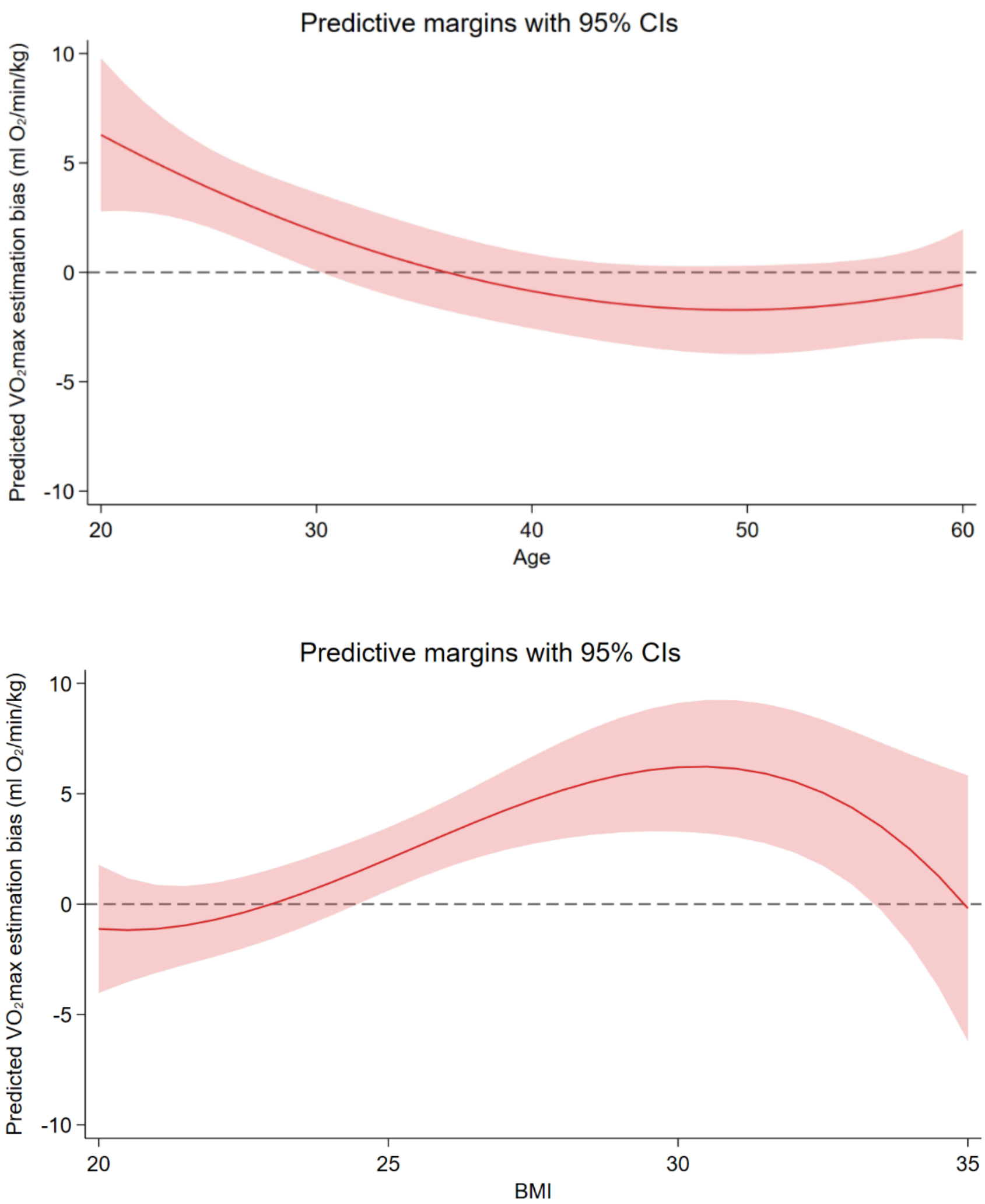

Supplementary Figure 3

Framework structure for estimating fitness from exercise heart rate response. This flowchart visualises the automated computational workflow. The framework ingests measured exercise data (yellow region), passes it through the mathematical model (orange region), and iteratively optimises the results (blue region). Rectangles represent static data inputs, while rounded rectangles represent active computational processes. The two-way arrows indicate the machine-learning loop (Particle Swarm Optimisation), which continuously refines the model parameters until the simulated heart rate matches the measured heart rate, while remaining physiologically plausible. The final output yields the patient's individualised $\dot{V}O_2max$, HRmax, and metabolic efficiency coefficients.

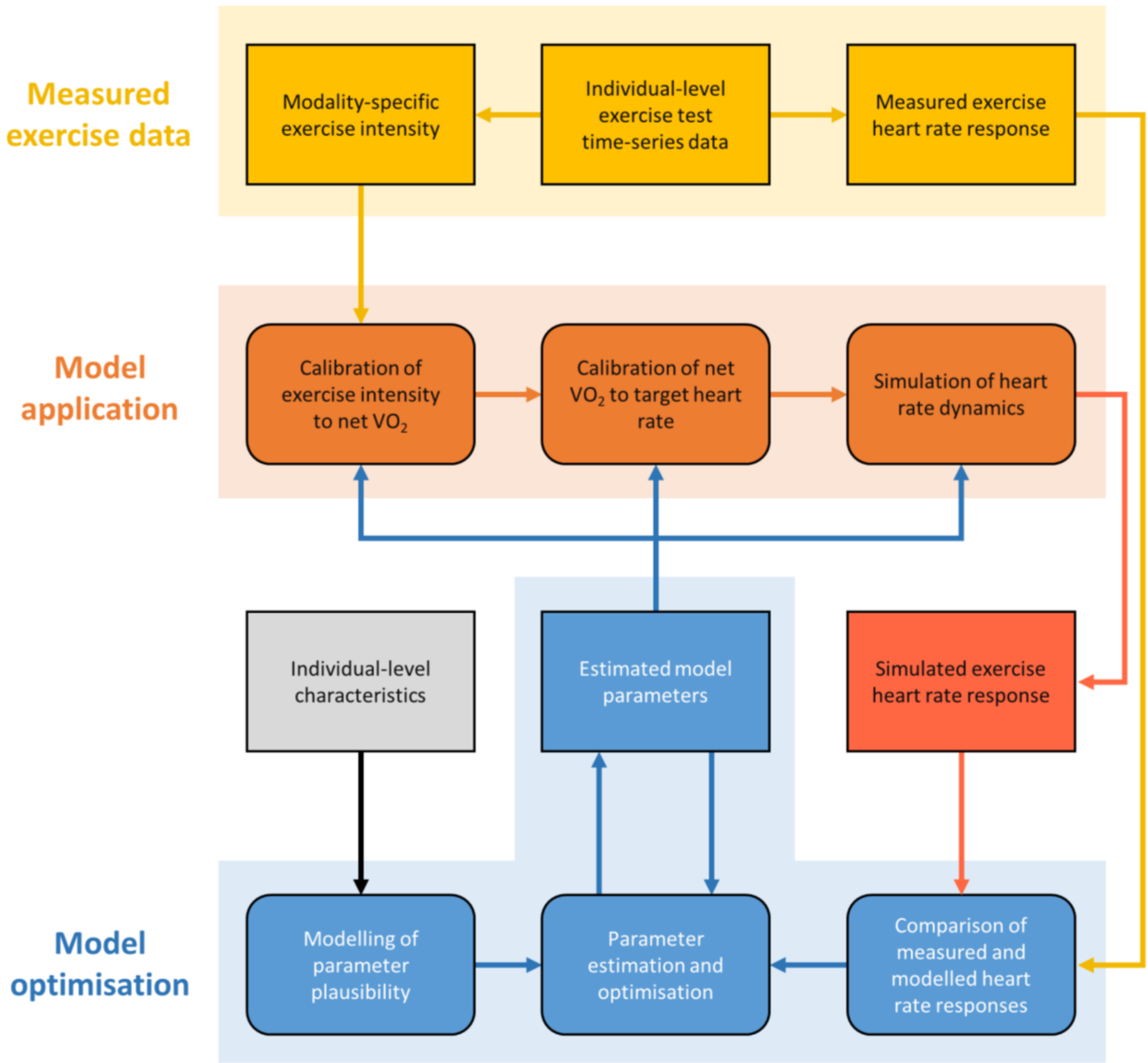

Supplementary Figure 4

Exemplar plots demonstrating the framework processing a single individual's data across diverse exercise tests. **A.** The raw modality-specific external work rate (e.g., treadmill speed/grade or cycle wattage) inputted into the framework. **B.** The framework calibrates this mechanical work into an individualised metabolic demand represented as net VO_2. **C.** The framework dynamically models the inherently lagged heart rate response to this metabolic demand. The algorithm continuously adjusts the patient's estimated fitness parameters until the simulated heart rate trajectory (modelled line) optimally aligns with their actual measured heart rate (data points) throughout the duration of the test.

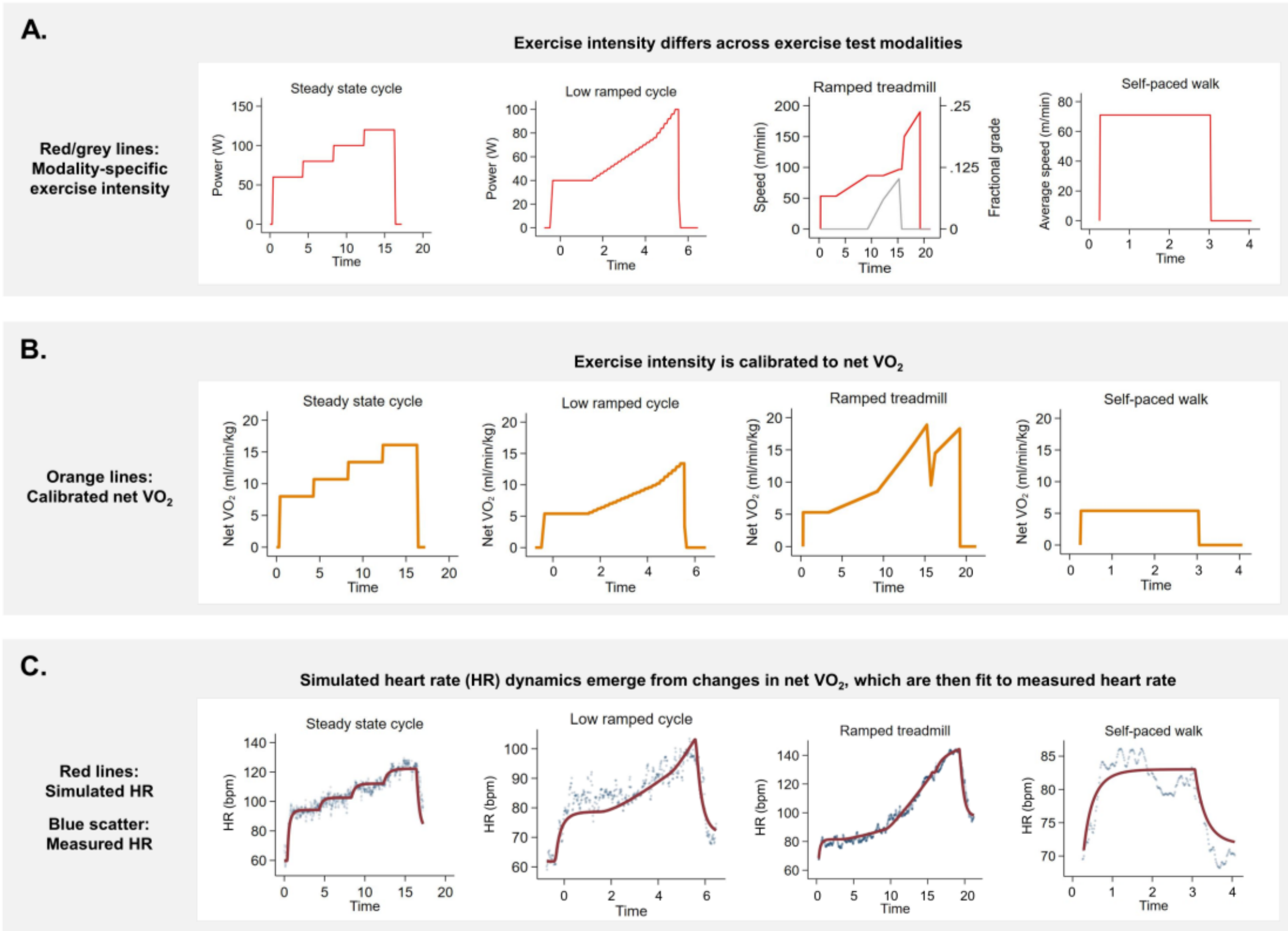

Supplementary Figure 5

Conceptual overview of Particle Swarm Optimization (PSO). This illustration shows how the algorithm searches for a participant's fitness parameters. Each "particle" (black dot) represents a candidate set of physiological parameters (e.g., a specific guess for the participant's $\dot{V}O_2$max and metabolic coefficients). At 'Iteration # 0', these candidate profiles are randomly distributed across the mathematically possible parameter space. Guided by the objective function, the particles move through the space, adjusting their trajectories based on which parameter combinations best fit the participant's measured heart rate. By 'Iteration # N', the swarm converges on the optimal solution (star), representing the most accurate and physiologically plausible fitness estimate for that individual.

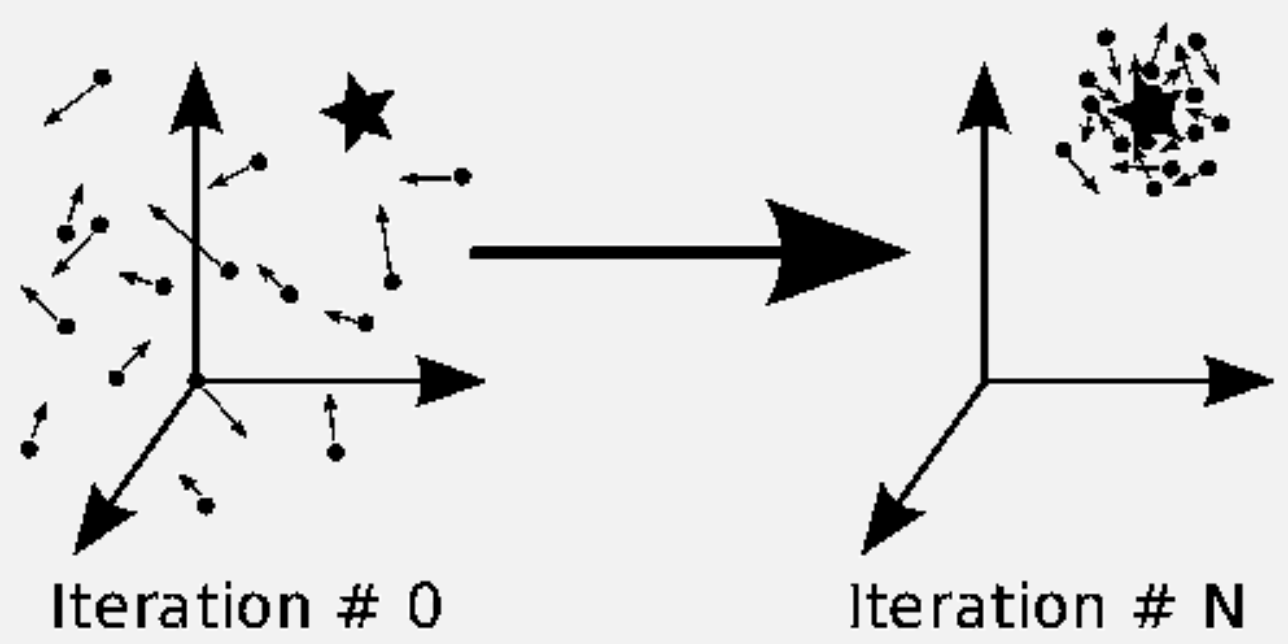

Step 1. Initialise particles with random positions (parameter values).
- pBest (Personal Best): The best position each particle has achieved so far.
- gBest (Global Best): The best position achieved by any particle in the swarm.

Step 2. For each particle:
a. Evaluate the objective function at its position.
b. Update pBest if the current position has a better objective function value.

Step 3. Update gBest with the best position found in the swarm based on the objective function.

Step 4. For each particle:
a. Adjust velocity based on its pBest and the swarm's gBest.
b. Update position (parameter values) using this velocity.

Step 5. If stopping criteria are met (e.g., maximum number of iterations, objective function threshold reached), stop; else, repeat from Step 2.

Supplementary Figure 6

Conceptual figure illustrating the Mahalanobis distance penalty for physiological plausibility. While PSO ensures the estimated parameters match the measured heart rate, this penalty encourages the resulting estimates to be physiologically plausible. Parameters X1 and X2 (e.g., estimated $\dot{V}O_2max$ and metabolic cost) are modelled as normal distributions intersecting at a centroid (red star), which represents the expected population norm. The elliptical dashed lines represent contours of equal Mahalanobis distance, accounting for the covariance between variables. Candidate estimates (black dots) that fall closer to the centroid have a lower distance, indicating higher physiological plausibility. This prevents the framework from assigning extreme or biologically contradictory physiological profiles to a participant.

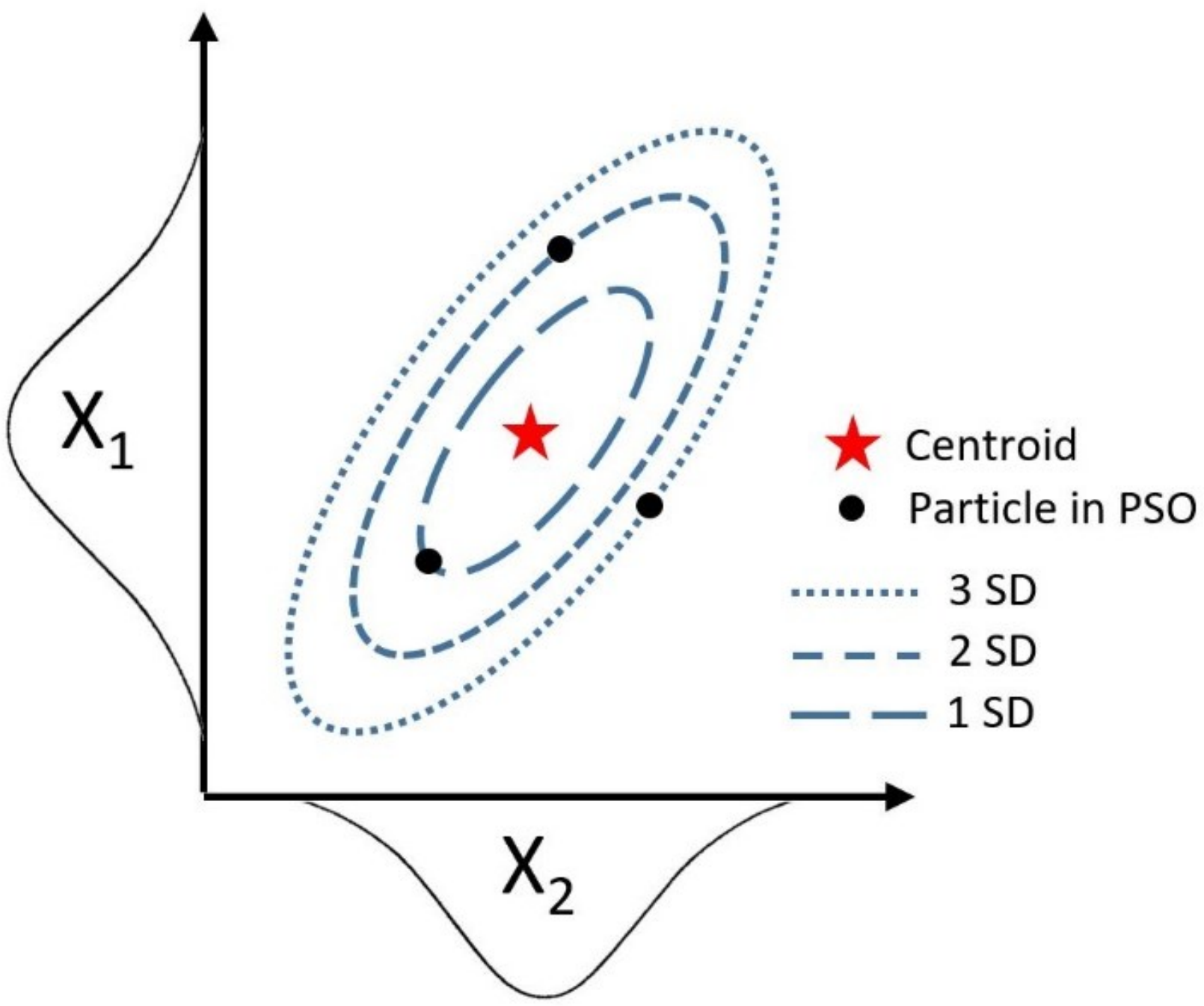

Supplement: Supplementary file 1 [file 44437_2026_4_MOESM1_ESM.pdf]
